# *Tripterygium wilfordii* Hook F versus conventional synthetic disease-modifying anti-rheumatic drugs as monotherapy for rheumatoid arthritis: a systematic review and network meta-analysis

**DOI:** 10.1186/s12906-016-1194-x

**Published:** 2016-07-13

**Authors:** Hai-Long Wang, Quan Jiang, Xing-Hua Feng, Hua-Dong Zhang, Lin Ge, Cheng-Gui Luo, Xun Gong, Bo Li

**Affiliations:** Division of Rheumatology, Guang An Men Hospital, China Academy of Chinese Medical Sciences, Beijing, 100053 China

**Keywords:** *Tripterygium wilfordii* Hook F, Conventional synthetic DMARDs, Systematic review, Network meta-analysis, Rheumatoid arthritis

## Abstract

**Background:**

*Tripterygium wilfordii* Hook F (TwHF), a medicinal plant that has been widely used in Chinese traditional medicine, is proven effective for treating rheumatoid arthritis (RA), but its clinical efficacy and safety remain largely undefined in comparison with conventional synthetic disease modifying anti-rheumatic drugs (DMARDs).

**Methods:**

PubMed, Embase, Cochrane Library, CNKI, VIP, CBM, and WanFang Databases. Endpoints were ACR 20, 50, and 70, and the number of withdrawals due to adverse events. Initially, traditional pairwise meta-analysis was performed by using a random-effects model. Then, we performed network meta-analysis to compare different therapies by using frequentist approach.

**Results:**

A total of 22 trials (5255 participants) were identified. By direct comparison, TwHF was superior to sulphasalazine according to ACR 20, 50 and 70. TwHF was superior to placebo according to ACR 20 and 50. By indirect comparisons, TwHF was superior to methotrexate, leflunomide, sulphasalazine, tacrolimus, minocycline and placebo according to ACR 20. Ranking by the Surface under the Cumulative Ranking curve (SUCRA) values showed that TwHF had the greatest probability for being the best treatment option according to ACR 20 (92.0 %) and ACR 50 (81.3 %), and the highest probability to be in the second (57.8 %) ranking position after leflunomide (69.6 %) according to ACR 70. By both direct and indirect comparisons, TwHF caused no more significant withdrawals than the placebo. The SUCRA values showed that TwHF had the highest probability to rank sixth (26.7 %) after the placebo (45.6 %) in causing withdrawals.

**Conclusions:**

Our data suggest that TwHF is effective and safe in the treatment of RA and has better clinical efficacy in terms of ACR 20 and 50 than existing conventional synthetic DMARDs. In the absence of head-to-head treatment comparison, the confidence in these estimates is low. Future comparative efficacy studies are warranted.

**Electronic supplementary material:**

The online version of this article (doi:10.1186/s12906-016-1194-x) contains supplementary material, which is available to authorized users.

## Background

Rheumatoid arthritis (RA) is an autoimmune disease of unknown etiology that is characterized by pain, stiffness, and swelling of peripheral joints. If uncontrolled, it may result in progressive joint destruction, deformity, disability, and increased mortality. Given the chronic nature of RA, it is important to obtain evidence of long-term success of therapy with disease modifying anti-rheumatic drugs (DMARDs). There are three major classes of DMARDs including conventional synthetic, targeted synthetic DMARDs, and biological agents [1]. These DMARDs may prevent or reduce joint damage, and help to maintain regular structure and function of the joint. As first-line treatment in RA, conventional synthetic DMARDs are often used as monotherapy in the early course of the illness [2].

*Tripterygium wilfordii* Hook F (TwHF), also known as Lei Gong Teng or thunder god vine in traditional Chinese medicine, has been used to treat RA [3] as a DMARD for many years in China [4, 5]. There are approximately 380 metabolites been identified from extracts of TwHF. Three of the extracts dominate the chemical profile and the medicinal chemistry of TwHF, including triptolide, tripdiolide and triptonide [6]. The extracts of TwHF have shown anti-inflammatory and immunosuppressive activities both in vivo and in vitro studies [7, 8]. A randomized double blind, placebo controlled trial showed that RA patients who had received six weeks of topical *Tripterygium wilfordii* attained a markedly higher modified American College of Rheumatology Criterion of 20 % (ACR 20) response rate compared to those who had taken the placebo [9]. Another randomized, controlled trial (RCT) demonstrated that RA patients who received 24 weeks of TwHF extract achieved significantly greater ACR 20 response rate than those who were treated with sulfasalazine [10]. However, data from these studies and others could not be pooled due to differing interventions, comparisons and outcome measures [10–12]. Since head-to-head comparison studies are sparse, traditional pairwise meta-analysis could not provide an answer to which intervention is superior to others [13, 14]. Network meta-analysis is a recently developed method that could deal with evidence from direct comparisons (from studies directly comparing interventions) as well as indirect comparisons (two treatments derived via a common comparator) [15–17]. In this systematic review and network meta-analysis, we aimed to evaluate the efficacy and safety of TwHF compared with conventional synthetic DMARDs with ACR 20, American College of Rheumatology Criterion of 50 % (ACR 50), and American College of Rheumatology Criterion of 70 % (ACR 70) as the primary outcomes and safety as the secondary outcome.

## Methods

### Data sources and searches

Our study protocol was registered on PROSPERO (CRD42014015179). We searched PubMed, EMBASE, and the Cochrane Library. In addition, we searched the Chinese databases, including the CNKI Database, VIP, CBM, and WanFang Database. All the databases were searched from their date of inception to the latest issue (Jan, 2015).

Searches included a combination of free-text and Medline Subject Headings (MeSH) terms for ‘disease terms’ with ‘drug names’, and were limited to published ‘human’ RCTs. For the English databases, we used free text terms, such as “*Tripterygium wilfordii* Hook F”, methotrexate, leflunomide, sulphasalazine, hydroxychloroquine, cyclosporine A, azathioprine, cyclophosphamide, mycophenolate mofetil, tacrolimus (FK506), intramuscular gold, auranofin, minocycline, *D*-penicillamin, chlorambucil, “rheumatoid arthritis”, and “randomized controlled trials”. For the Chinese databases, free texts were used, such as “lei gong teng” and “lei feng shi guan jie yan (rheumatoid arthritis)”. A filter for clinical trials was applied. To collect an adequate number of trials, we also searched the reference lists of relevant publications to identify additional studies.

### Study selection

Only RCTs comparing conventional synthetic DMARDs as monotherapy were included. The full-text publications were assessed for inclusion according to the following criteria: (1) the subjects took TwHF extract alone or conventional synthetic DMARDs for at least 12 weeks; (2) the study was a RCT with a parallel or crossover design; (3) the treatment was used as an active therapeutic intervention; (4) the enrolled subjects were diagnosed with RA according to the 1987 Guidelines of the American Rheumatology Association [18] or the 2010 ACR/ European League against Rheumatism (EULAR) Criteria [19]; (5) the clinical endpoints were ACR 20, 50, and 70 [20], and the safety endpoint was withdrawal due to drug-emergent adverse events. TwHF, in this review, mainly refers to the tripterygium glycoside tablet and tripterygium tablet, two root preparations of TwHF that have shown therapeutic promise [21, 22]. Studies using TwHF-containing herbs or other herbal extracts were excluded. The analyses of outcomes were conducted on an intent-to-treat (ITT) basis, or modified ITT (number actually receiving treatment at baseline) if the number randomized to treatment was not reported. The eligibility of studies for the inclusion criteria was assessed independently by four reviewers (C-GL, LG, XG, and BL).

### Data extraction and quality assessment

Two investigators (C-GL and XG) independently extracted and entered the following information into a database: study design, patient characteristics, interventions, comparisons, and outcomes. When relevant information on design or outcomes was unclear, or when doubt existed about duplicate publications, we contacted the original authors for clarifications. Two investigators (H-DZ and H-LW) independently evaluated the methodological quality of eligible trials by using the Cochrane Collaboration’s tool for assessing risk of bias [23] (random sequence generation, allocation concealment, blinding of participants and personnel, blinding of outcome assessment, incomplete outcome data, selective reporting, and other sources of bias). Disagreement between the two authors was resolved by discussion. When disagreement persisted, two other senior investigators (QJ and X-HF) were consulted to attain consensus.

### Data synthesis and analysis

The primary outcome of this analysis was treatment response in terms of ACR 20, 50, or 70 defined as a 20 %, 50 %, or 70 % improvement in tender and swollen joints and the same level of improvement in three of the five following variables: patient and physician global assessment of overall disease activity; patient evaluation of pain; a score of physical disability; blood acute-phase reactants. The secondary outcome for safety was withdrawal of patients due to drug-emergent adverse events.

### Network meta-analysis

Results are reported as odds ratios (ORs) with 95 % confidence intervals (CI) for all comparisons of interventions. Initially, traditional pairwise meta-analysis was performed by using a random-effects model. Then, we performed network meta-analysis to compare different therapies by using frequentist approach [24, 25]. We included multi-arm trials in the analysis by breaking multi-arm trial into separate two-arm trials. We employed a multivariate random-effects meta-analysis model for each outcome separately, combining direct evidence for each comparison [26, 27].

For each ‘loop’ of treatment comparisons from three or more independent sources and for each outcome, we computed the difference between estimates from direct and indirect evidence on the log OR scale. Inconsistency was defined as disagreement between direct and indirect evidence with a 95 % CI excluding 0.

For each outcome, we estimated the probability that which intervention was the best for each outcome, the second best, and the third best and so on, from the rank orderings of the treatments at each interaction. Rank 1 indicated the highest efficacy and Rank 8 means lowest of a treatment. These ranking probabilities were used to calculate the Surface under the Cumulative Ranking curve (SUCRA), which is expressed as percentage (100 % for the best intervention and 0 % for the worst intervention and approximately 50 % for equivalent interventions) [28]. The detailed methods of network meta-analysis in the study are presented as Appendix Information (Additional file 1).

### Funnel plot and publication bias

The difference between the observed effect size and comparison-specific summary effect for each study was calculated. This variable was then regressed on the standard error (SE), thus adding a simple linear regression line in the funnel plot. This method could help visually determine if there is a publication bias in the results between small and large studies.

We performed traditional and network meta-analysis by using Stata software (version 12.0, the StataCorp, College Station, Texas, USA).

## Results

**Fig. 1:**
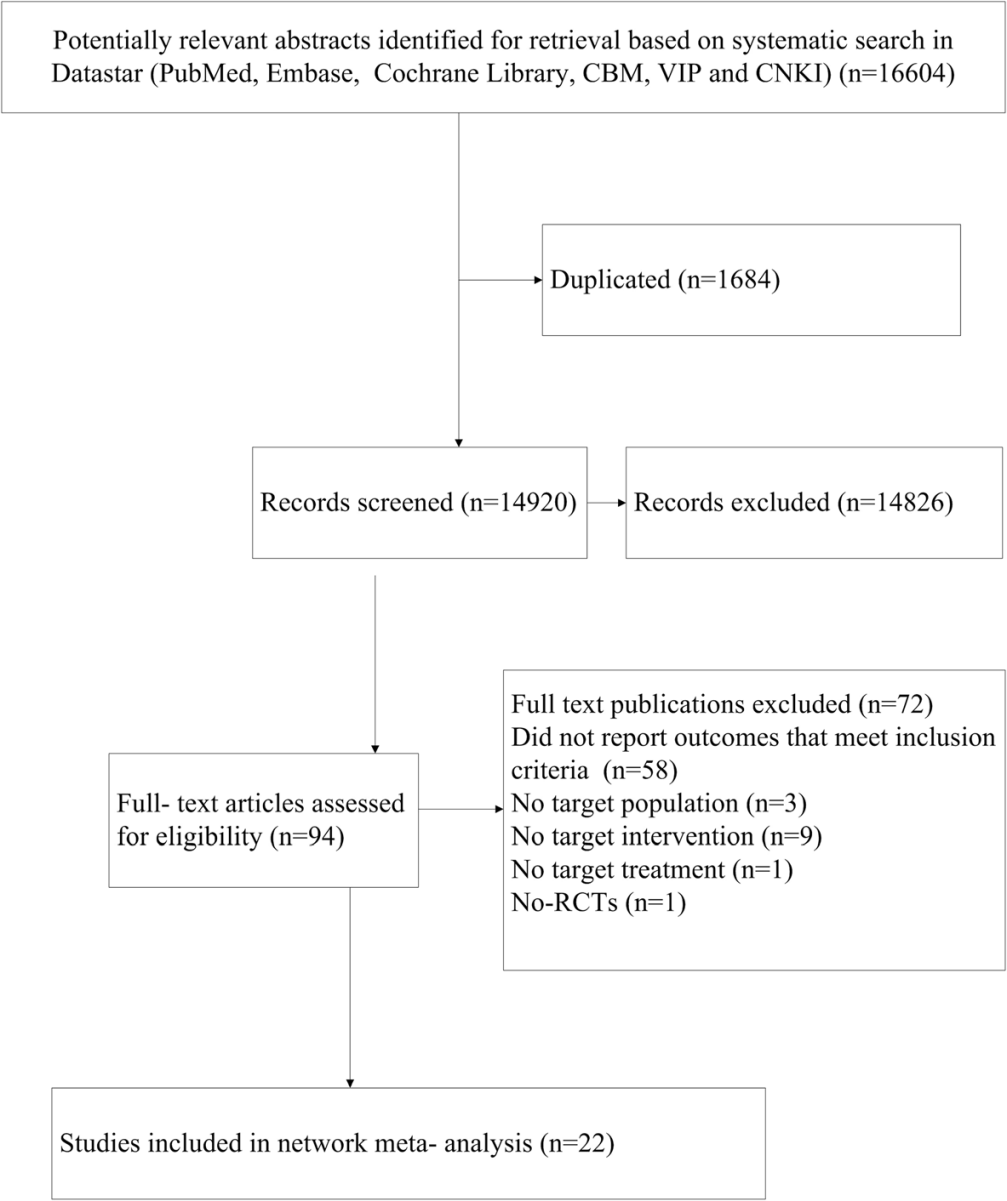
The flow chart of studies considered for inclusion

The flow chart of studies considered for inclusion is shown in Fig. 1. On the basis of the title and abstract, 94 publications were selected and analyzed in full-text versions. Additional file 2: Figure S1A through D shows the network of all treatment comparisons analyzed according to ACR 20, 50, 70 and withdrawal. Eventually, 22 publications were included in the systematic review. All reviews followed the methods in the Cochrane Handbook, including standardized searches, inclusion criteria and outcomes.

### Characteristics of included studies

We found 22 eligible studies from 22 publications, which enrolled a total of 5255 patients and evaluated 6 different conventional synthetic DMARDs.

Additional file 3: Table S1 summarizes the clinical and methodological characteristics as well as the main outcomes of each trial. Of the 22 trials included, TwHF was studied in 3 trials. Combined sample size in the TwHF arms was 149. The risk of bias assessments for the included trials is illustrated in Additional file 2: Figure S2 and Figure S3. Most of the evidence was of moderate-to-good quality. All 22 RCTs mentioned the word “randomization”. Over half of the studies did not report adequate information about allocation sequence generation and allocation sequence concealment. Unblinded designs were used in half of the trials included.

### Clinical efficacy

ACR 20 was reported in 22 trials (5255 participants) (Additional file 3: Table S1), covering all the eight interventions of interest (Table 1). Based on direct comparison alone, TwHF was statistically significantly superior to sulphasalazine and placebo (OR from 3.81 to 36.77, 95 % CI excluded 1). Based on indirect comparisons alone, TwHF was statistically significantly superior to methotrexate, leflunomide, sulphasalazine, FK506, minocycline and placebo (estimated ORs ranged from 11.76 to 33.33, 95 % CI excluded 1). The ranking information for all interventions by the SUCRA values is shown in Additional file 2: Figure S4A and Table 2. According to the ranking probabilities, TwHF had the greatest probability (92.0 %) for being the best treatment option according to ACR 20, followed by leflunomide (63.4 %) and sulphasalazine (62.4 %) versus the placebo (6.6 %).

**Table 1:** Odds ratio (95 % CI) by direct comparison (left lower part) and network meta- analysis (right upper part) based on ACR20

TwHF	15.56 (1.63–148.5)	12.09 (1.31–111.5)	11.76 (1.21–113.9)	0.89 (0.00–2.18e+28)	14.77 (1.59–137.2)	17.04 (1.76–165.3)	33.33 (4.17–100.0)
1.49 (0.73–3.08)	MTX	0.78 (0.43–1.41)	0.76 (0.35–1.61)	0.06 (0.00–1.35e+27)	0.95 (0.51–1.76)	1.10 (0.51–2.34)	2.38 (1.45–4.00)
–	0.94 (0.79–1.10)	LEF	0.97 (0.51–1.86)	0.07 (0.00–1.74e+27)	1.22 (0.76–1.97)	1.41 (0.74–2.70)	3.13 (2.27–4.17)
3.81(1.79–8.09)	0.89 (0.57–1.39)	1.25 (0.97–1.61)	SSZ	0.08 (0.00–1.79e+27)	1.26 (0.64–2.46)	1.45 (0.65–3.24)	3.23 (1.82–5.56)
–	–	0.47 (0.15–1.46)	–	CsA	16.52 (0.00–3.88e+29)	19.06 (0.00–4.48e+29)	50.00 (0.00–100.0)
–	–	–	–	–	FK506	1.15 (0.59–2.26)	2.56 (1.75–3.70)
–	–	–	–	–	–	MINO	2.22 (1.25–3.85)
36.77 (1.91–708.0)	2.41 (1.46–3.98)	3.03 (2.21–4.14)	3.18 (1.80–5.62)	–	2.38 (1.69–3.76)	2.19 (1.25–3.88)	Placebo

Results of direct comparisons are listed in the lower-left triangle, and the estimation is calculated as the row-defining treatment compared with the column-defining treatment. Results of network meta-analysis are listed in the upper-right triangle, and the estimation is calculated as the column-defining treatment compared with the row-defining treatment

*TwHF Tripterygium wilfordii* Hook F, *MTX* methotrexate, *LEF* leflunomide, *SSZ* sulphasalazine, *CsA* cyclosporine, *FK506* tacrolimus, *MINO* minocycline

**Table 2:** Ranking probability of DMARDs

Treatment	ACR20		ACR50		ACR70		Withdrawal	
	SUCRA	Rank	SUCRA	Rank	SUCRA	Rank	SUCRA	Rank
TwHF	0.920	1	0.813	1	0.578	2	0.267	6
MTX	0.418	6	0.611	3	0.454	6	0.158	7
LEF	0.634	2	0.673	2	0.696	1	0.758	2
SSZ	0.624	3	0.444	5	0.470	5	0.888	1
CsA	0.526	4	0.502	4	0.510	4	0.483	4
FK506	0.453	5	0.369	6	0.545	3	0.489	3
MINO	0.359	7	–	–	–	–	–	–
Placebo	0.066	8	0.087	7	0.247	7	0.456	5

*TwHF Tripterygium wilfordii* Hook F, *MTX* methotrexate, *LEF* leflunomide, *SSZ* sulphasalazine, *CsA* cyclosporine, *FK506* tacrolimus, *MINO* minocycline, *SUCRA* surface under the Cumulative Ranking curve

ACR 50 was reported in 13 trials (2590 participants), covering seven interventions of interest (Additional file 3: Table S2). Based on direct comparisons alone, TwHF was statistically significantly superior to sulphasalazine and placebo (OR from 9.67 to 21.97, 95 % CI excluded 1). Based on indirect comparisons alone, TwHF was statistically significantly superior to placebo (OR 11.11, 95 % CI excluded 1). Additional file 2: Figure S4B and Table 2 show the SUCRA values for all interventions. According to the ranking probabilities, TwHF had the greatest probability (81.3 %) to be the best treatment option according to ACR 50, followed by leflunomide (67.3 %), methotrexate (61.1 %), and cyclosporine A (50.2 %) versus the placebo (8.7 %).

ACR 70 was reported in 10 trials (2182 participants), covering seven interventions of interest (Additional file 3: Table S3). Based on direct comparisons alone, TwHF was statistically significantly superior to sulphasalazine (OR 12, 95 % CI excluded 1). Based on indirect comparisons alone, TwHF caused no more significant than other interventions. Additional file 2: Figure S4C and Table 2 show the ranking information for all interventions by the SUCRA values. The treatment of TwHF showed the highest probability to be in the second (57.8 %) ranking position after leflunomide (69.6 %) and followed by FK506 (54.5 %) and cyclosporine A (51.0 %) versus the placebo (24.7 %).

### Safety

The number of withdrawals was reported in 15 trials (3928 participants), covering seven interventions of interest (Table 3). Based on direct comparisons alone, TwHF caused no more significant withdrawals than the placebo (OR 0.37, 95 % CI included 1) while leflunomide, and sulphasalazine led to more withdrawals due to adverse events than the placebo (estimated ORs ranged from 3 to 3.32, 95 % CI excluded 1). Based on indirect comparisons alone, TwHF caused no more significant withdrawals than the placebo (OR 0.36, 95 % CI included 1) while leflunomide, and sulphasalazine led to more withdrawals due to adverse events than placebo (estimated ORs ranged from 1.92 to 3.33, 95 % CI excluded 1). The SUCRA values presented in Additional file 2: Figure S4D and Table 2 show the ranking information for all interventions. According to this rank probability, TwHF had the highest probability to be ranked at the sixth (26.7 %) after the placebo (45.6 %), while sulphasalazine had the greatest probability (88.8 %) for causing most withdrawals.

**Table 3:** Odds ratio (95 % CI) by direct comparison (left lower part) and network meta- analysis (right upper part) based on safety

TwHF	1.21 (0.10–14.26)	0.19 (0.02–2.07)	0.11 (0.01–1.36)	0.66 (0.00–1.64e+28)	0.35 (0.03–3.74)	0.36 (0.04–3.85)
0.32 (0.03–3.19)	MTX	0.16 (0.06–0.40)	0.09 (0.03–0.31)	0.55 (0.00–1.31e+28)	0.29 (0.11–0.73)	0.30 (0.14–0.68)
–	0.74 (0.49–1.10)	LEF	0.58 (0.20–1.66)	3.48 (0.00–8.25e+28)	1.82 (0.93–3.54)	1.92 (1.18–3.13)
0.39 (0.16–1.01)	0.71 (0.40–1.25)	0.93 (0.51–1.36)	SSZ	6.02 (0.00–1.43e+29)	3.15 (1.11–8.91)	3.33 (1.29–8.33)
–	–	2.06 (0.35–12.06)	–	CsA	0.52 (0.00–1.27e+33)	0.55 (0.00–100.0)
–	–	–	–	–	FK506	1.05 (0.67–1.67)
0.37 (0.01–9.98)	0.30 (0.14–0.68)	3.00 (1.87–4.83)	3.32 (1.30–8.45)	–	1.03 (0.65–1.61)	Placebo

Results of direct comparisons are listed in the lower-left triangle, and the estimation is calculated as the row-defining treatment compared with the column-defining treatment. Results of network meta-analysis are listed in the upper-right triangle, and the estimation is calculated as the column-defining treatment compared with the row-defining treatment

*TwHF Tripterygium wilfordii* Hook F, *MTX* methotrexate, *LEF* leflunomide, *SSZ* sulphasalazine, *CsA* cyclosporine, *FK506* tacrolimus, *MINO* minocycline

### Evaluating and presenting assumptions

#### Inconsistency check

Statistical inconsistency between direct and indirect comparisons was generally low for 4 outcomes (Additional file 2: Figures S5A through D). All loops were consistent because the 95 % CIs included 0 according to the forest plots, indicating that the direct estimation of the summary effect does not differentiate from the indirect estimation. The summary estimations of the network meta- analysis are relatively robust.

#### Estimated summary effects

We presented the mean effect sizes for the network estimates along with their CI and predictive intervals (PrI), all based on an assumption of a common heterogeneity variance (Additional file 2: Figures S6A through D). The plot indicates that TwHF appear more effective than methotrexate, leflunomide, FK506, and minocycline in ACR 20. The CI for TwHF vs sulphasalazine potentially change the interpretation of the findings compared with the PrI for ACR 20, since they extend across the line of OR = 1 when the PrI does not.

#### Funnel plot and publication bias

Funnel plots for ACR 20, 50, 70 and withdrawals are shown in Additional file 2: Figures S7A through D. Scatters in the 4 funnel plots were almost symmetrical visually, indicating that the publication bias in the results of these 4 outcomes between small and large studies was relatively low.

## Discussion

Various extracts of HwHF has been widely used in China for hundreds of years for various symptoms. The chloroform- methanol extract of the roots of TwHF has been investigated as a potential treatment for autoimmune diseases [12, 29–31]. Over the past 30 years, extracts of TwHF have become a standard therapy for rheumatoid arthritis in China. Recently, extracts of TwHF have been tested in the West and shown good efficacy [10, 32]. The results of the current study showed that TwHF is effective for treating active RA patients and is superior to placebo and sulphasalazine according to ACR 20, 50, and 70. TwHF is superior to conventional synthetic DMARDs such as methotrexate, leflunomide, sulphasalazine, FK506 and minocycline in clinical efficacy by ACR 20. Ranking by the SUCRA values showed that TwHF had the greatest probability for being the best treatment option according to ACR 20 and ACR 50, and the highest probability to be in the second ranking position after leflunomide according to ACR 70. More importantly, the result of the current analysis found that TwHF is safe, causing no more drug-emergent withdrawals than the placebo. Our findings suggest that this Chinese herbal remedy is both effective and safe in treating active RA, offering RA patients a potentially effective and safe alternative to conventional synthetic DMARDs whose efficacies are hampered by untoward effects.

Consistent with our findings, a recent systemic review also found that TwHF could be as effective as synthetic DMARDs in the treatment of RA [33]. The same study showed that the methodological quality of the studies included in their study was generally low and the approach the authors used cannot integrate all the evidence from several comparators. Network meta-analysis combines both direct and indirect evidence for multiple treatments to estimate interrelations across all treatments. To the best of our knowledge, our study represents the first employment of network meta-analysis for comparing different conventional synthetic DMARDs in treating RA. Using this approach, we were able to combine simultaneously all relevant evidence on conventional synthetic DMARDs in treating RA patients, even in the absence of direct comparative evidence for some treatment pairs, encompassing four efficacy and safety outcomes.

Since more evidence of direct active comparisons is reported, our network meta-analysis provides a useful and complete picture for the propensity of anti-rheumatic treatment. This statistical technique not only includes the results of direct comparisons but also incorporates indirect comparisons, particularly for TwHF versus leflunomide, cyclosporine A, FK506, and minocycline, which have been rarely compared in head to head trials. Based on the data from 22 trials including 5255 patients randomly assigned to eight different intervention regimes for RA, we found substantial uncertainty about the relative efficacy and safety of different interventions in respect of the studied outcomes. As there were differences in treatment efficacy and safety among those conventional synthetic DMARDs, we also reported the probabilities of ranking for these treatments, providing additional evidence for the comparative efficacy of these conventional synthetic DMARDs.

Our findings have several limitations. Firstly, trials investigating combination therapies were excluded in our study. Although combination therapy is common in practice, the large number of possible therapy combinations and scarcity of trials comparing these treatments prompted us to restrict our analysis to monotherapies for RA. Secondly, variations of drug ingredients or doses may contribute to variations in study outcomes. However, treating different doses of the same drug or different drug ingredients as different treatment regiments would not be feasible owing to insufficient patient numbers and events of forming a well-connected network, so we only evaluated treatment effects and safety of major drug classes. Thirdly, most included studies did not mention the details of randomization and concealed allocation, and some of them were of small sample size. Due to the high risk of bias in these studies, the results of our network meta-analysis might be biased. Finally, as our review aimed to include only English and Chinese full-length publications published in peer-reviewed journals, there is a potential for publication bias.

## Conclusions

Our network meta-analysis, for the first time, provides a useful and complete picture of the associations between TwHF, conventional synthetic DMARDs and placebo according to ACR 20, 50, 70 and safety. We found the efficacy of TwHF was statistically significantly superior to placebo according to ACR 20 and 50. TwHF was statistically significantly superior to sulphasalazine according to ACR 20, 50 and 70. Clinically important information from high-quality randomized trials is still needed for decision-making regarding primary treatment options for RA using TwHF. More trial evidence is required to reduce uncertainty. TwHF may be used as the first line DMARDs agent in the treatment of RA. Network meta-analysis may be useful to optimize the power of evidence studies once data from new randomized controlled studies in this field are published in the future.

## Abbreviations

ACR 20, American College of Rheumatology Criterion of 20 %; ACR 50, American College of Rheumatology Criterion of 50 %; ACR 70, American College of Rheumatology Criterion of 70 %; CI, confidence intervals; DMARDs, Disease-modifying Anti-rheumatic Drugs; EULAR, European league against rheumatism; ITT, intent-to-treat; OR, odds ratio; PrI, predictive intervals; RA, Rheumatoid arthritis; RCT, randomized, controlled trial; SE, standard error; SUCRA, Surface under the Cumulative Ranking curve; TwHF, *Tripterygium wilfordii* Hook F.

## Additional files

Additional file 1: Methods of Network meta-analysis in the study. (DOCX 22 kb) Additional file 2: Figure S1. Network of eligible treatment comparisons for outcomes of ACR 20 (A), 50 (B), 70 (C) and withdrawal (D). **Figure S2.** Methodological quality graph: review authors’ judgments about each methodological quality item presented as percentages across all included studies. **Figure S3.** Methodological quality summary: review authors’ judgments about each methodological quality item for each included study. **Figure S4.** Plot of the surface under the cumulative ranking curves for all treatments on ACR 20 (A), ACR 50 (B), ACR 70 (C), and safety (D). **Figure S5.** Plots of inconsistency check for all closed loops in the network on ACR 20 (A), ACR 50 (B), ACR 70 (C), and safety (D). **Figure S6.** Interval plots of results of meta-analysis comparing different interventions in ACR 20 (A), 50 (B), 70 (C) and withdrawal (D). **Figure S7.** Comparison- adjusted funnel plot for ACR 20 (A), ACR 50 (B), ACR 70 (C), and safety (D). (DOCX 5744 kb) Additional file 3: Table S1. Baseline characteristics of included trials (Additional file 4). **Table S2.** ACR 50 by direct comparisons and network meta-analysis. **Table S3.** ACR 70 by direct comparisons and network meta-analysis. (DOCX 32 kb) Additional file 4: Appendix Information Methods of network meta-analysis in the study. (DOC 34 kb)
