# Supplementary material for: Tripterygium wilfordii Hook F versus conventional synthetic disease-modifying anti-rheumatic drugs as monotherapy for rheumatoid arthritis: a systematic review and network meta-analysis
Source: BMC Complement Altern Med. 2016 Jul 13;16:215. doi: 10.1186/s12906-016-1194-x (PMC4944439; doi:10.1186/s12906-016-1194-x)
Supplement: Additional file 1: — Methods of Network meta-analysis in the study. (DOCX 22 kb) [file 12906_2016_1194_MOESM1_ESM.docx]

**Methods of Network meta-analysis in the study**

Initially, we performed traditional meta-analysis for studies that directly compared different treatment arms, and a random-effects model was employed for each outcome. Then, we performed network meta-analysis (NMA) to compare different therapies. We employed a multivariate random-effects meta-analysis model for each outcome separately, combining direct evidence for each comparison [[1-4](#_ENREF_1)]. With indirect evidence [The following is an example of a network analysis. An initial trial compares drug A to drug C. A different trial studying the same patient population compares drug B to drug C. Assume that drug A is found to be superior to drug C in the first trial. Assume drug B is found to be equivalent to drug C in a second trial. Network analysis then, allows one to potentially say statistically that drug A is also superior to drug B for this particular patient population. (Since drug A is better than drug C, and drug B is equivalent to drug C, then drug A is also better to drug B even though it was not directly tested against drug B.)]. The model accounts explicitly for the binary nature of each outcome using a binomial likelihood function; Allows for heterogeneity of treatment effects between trials of the same comparison (assuming the same amount of heterogeneity for each comparison, irrespective of how many trials address it) and enforces an underlying relationship between direct and indirect evidence for a particular comparison, assuming these are consistent between the two sources. For each ‘loop’ of treatment comparisons from three or more independent sources and for each outcome, we computed the difference between estimates from direct and indirect evidence on the log OR scale. This provides a measure of inconsistency between the different sources. The STATA command mvmeta makes NMA possible within a frequentist setting and properly accounts for correlations between effect sizes from multi-arm studies [[5](#_ENREF_5), [6](#_ENREF_6)]. By using this command we can perform a multivariate meta-regression because of covariates are allowed.

For each outcome, we estimated the probability that which intervention was superior to all others, the second best, and the third best and so on, from the rank orderings of the treatments at each interaction. Rank 1 indicated the highest efficacy and Rank 8 means lowest of a treatment. (using 1000 reps) In this section, we used a Bayesian scheme to sample from the posterior distributions of the estimated parameters and see which linear predictor is the best, and thus comes up with the relative probabilities. These ranking probabilities were used to calculate the Surface under the Cumulative Ranking curve (SUCRA), which is expressed as percentage (100% for the best intervention and 0% for the worst intervention and approximately 50% for equivalent interventions).

**References**

1. White IR, Barrett JK, Jackson D, Higgins JP. Consistency and inconsistency in network meta-analysis: model estimation using multivariate meta-regression. [Res Synth Methods](http://www.ncbi.nlm.nih.gov/pubmed/?term=Consistency+and+inconsistency+in+network+meta-analysis%3A+model+estimation+using+multivariate+meta-regression.). 2012;3:111-25.

2. Higgins JP, Jackson D, Barrett JK, Lu G, Ades AE, White IR. Consistency and inconsistency in network meta-analysis: concepts and models for multi-arm studies. [Res Synth Methods](http://www.ncbi.nlm.nih.gov/pubmed/?term=Consistency+and+inconsistency+in+network+meta-analysis%3A+model+estimation+using+multivariate+meta-regression.). 2012;3:98-110.

3. Jackson D, White IR, Riley RD. A matrix-based method of moments for fitting the multivariate random effects model for meta-analysis and meta-regression. Biom J. 2013;55:231-45.

4. Chaimani A, Higgins JP, Mavridis D, Spyridonos P, Salanti G. Graphical tools for network meta-analysis in STATA. PloS one. 2013;8:e76654.

5. White IR. Multivariate random-effects meta-analysis. Stata Journal 2009;9:40-56.

6. White IR. Multivariate random-effects meta-regression:Updates to mvmeta. Stata Journal 2011;11:255-70.
