# Supplementary material for: Tripterygium wilfordii Hook F versus conventional synthetic disease-modifying anti-rheumatic drugs as monotherapy for rheumatoid arthritis: a systematic review and network meta-analysis
Source: BMC Complement Altern Med. 2016 Jul 13;16:215. doi: 10.1186/s12906-016-1194-x (PMC4944439; doi:10.1186/s12906-016-1194-x)
Supplement: Additional file 2: Figure S1. — Network of eligible treatment comparisons for outcomes of ACR 20 (A), 50 (B), 70 (C) and withdrawal (D). Figure S2. Methodological quality graph: review authors’ judgments about each methodological quality item presented as percentages across all included studies. Figure S3. Methodological quality summary: review authors’ judgments about each methodological quality item for each included study. Figure S4. Plot of the surface under the cumulative ranking curves for all treatments on ACR 20 (A), ACR 50 (B), ACR 70 (C), and safety (D). Figure S5. Plots of inconsistency check for all closed loops in the network on ACR 20 (A), ACR 50 (B), ACR 70 (C), and safety (D). Figure S6. Interval plots of results of meta-analysis comparing different interventions in ACR 20 (A), 50 (B), 70 (C) and withdrawal (D). Figure S7. Comparison- adjusted funnel plot for ACR 20 (A), ACR 50 (B), ACR 70 (C), and safety (D). (DOCX 5744 kb) [file 12906_2016_1194_MOESM2_ESM.docx]

**Figure Legends**

**Figure S1** Network of eligible treatment comparisons for outcomes of ACR 20 (A), 50 (B), 70 (C) and withdrawal (D). Lines connect the interventions that have been studied in head-to-head (direct) comparisons in the eligible randomized controlled trials for each pairwise comparison and the size of every node is proportional to the number of randomized participants (sample size). The yellow lines represent trials with risk of allocation concealment. TwHF: *Tripterygium wilfordii* Hook F, MTX: methotrexate, LEF: leflunomide, SSZ: sulphasalazine, CsA: cyclosporine, FK506: tacrolimus, and MINO: minocycline.

**Figure S2** Methodological quality graph: review authors’ judgments about each methodological quality item presented as percentages across all included studies.

**Figure S3** Methodological quality summary: review authors’ judgments about each methodological quality item for each included study.

**Figure S4** Plot of the surface under the cumulative ranking curves for all treatments on ACR 20 (A), ACR 50 (B), ACR 70 (C), and safety (D). TwHF: *Tripterygium wilfordii* Hook F, MTX: methotrexate, LEF: leflunomide, SSZ: sulphasalazine, CsA: cyclosporine, FK506: tacrolimus, and MINO: minocycline.

**Figure S5** Plots of inconsistency check for all closed loops in the network on ACR 20 (A), ACR 50 (B), ACR 70 (C), and safety (D). All loops were consistent because the 95% CIs included 0 according to the forest plots, indicating that the direct estimation of the summary effect does not differentiate from the indirect estimation. TwHF: *Tripterygium wilfordii* Hook F, MTX: methotrexate, LEF: leflunomide, SSZ: sulphasalazine, CsA: cyclosporine, FK506: tacrolimus, and MINO: minocycline.

**Figure S6** Interval plots of results of meta-analysis comparing different interventions in ACR 20(A), 50 (B), 70 (C) and withdrawal (D). TwHF: *Tripterygium wilfordii* Hook F, MTX: methotrexate, LEF: leflunomide, SSZ: sulphasalazine, CsA: cyclosporine, FK506: tacrolimus, and MINO: minocycline.

**Figure S7** Comparison- adjusted funnel plot for ACR 20 (A), ACR 50 (B), ACR 70 (C), and safety(D). TwHF: *Tripterygium wilfordii* Hook F, MTX: methotrexate, LEF: leflunomide, SSZ: sulphasalazine, CsA: cyclosporine, FK506: tacrolimus, and MINO: minocycline.


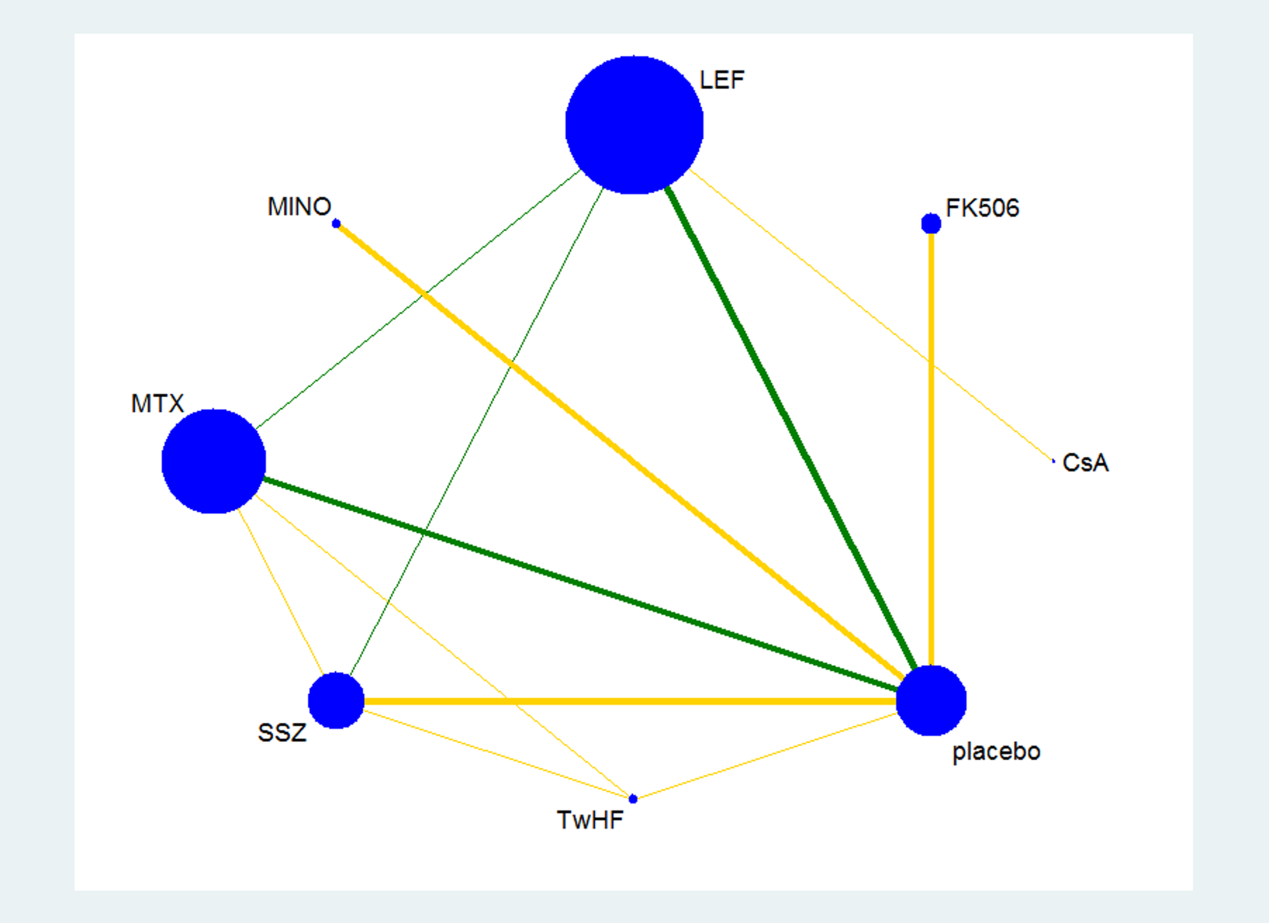


**Figure S1A**

**
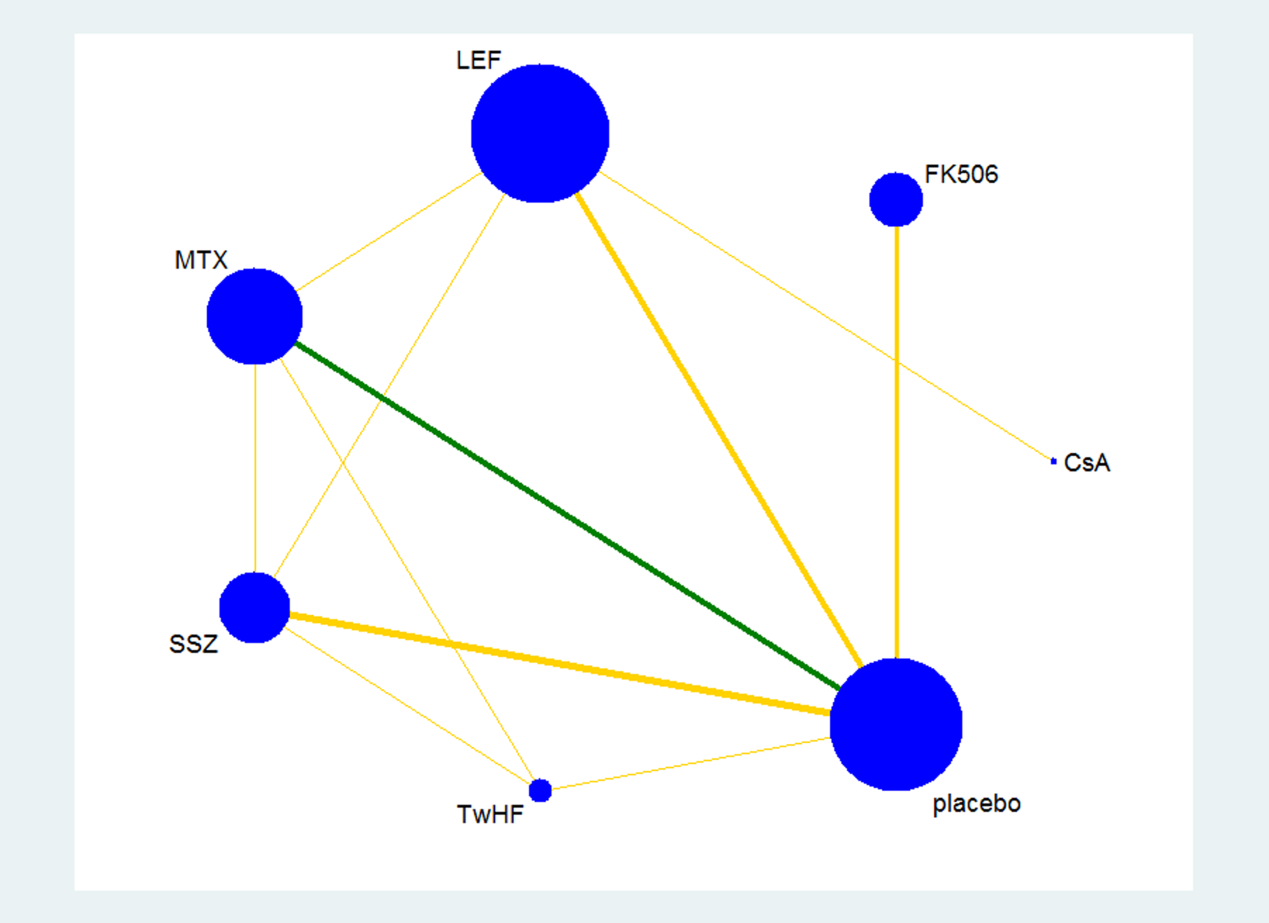
**

**Figure S1B**

**
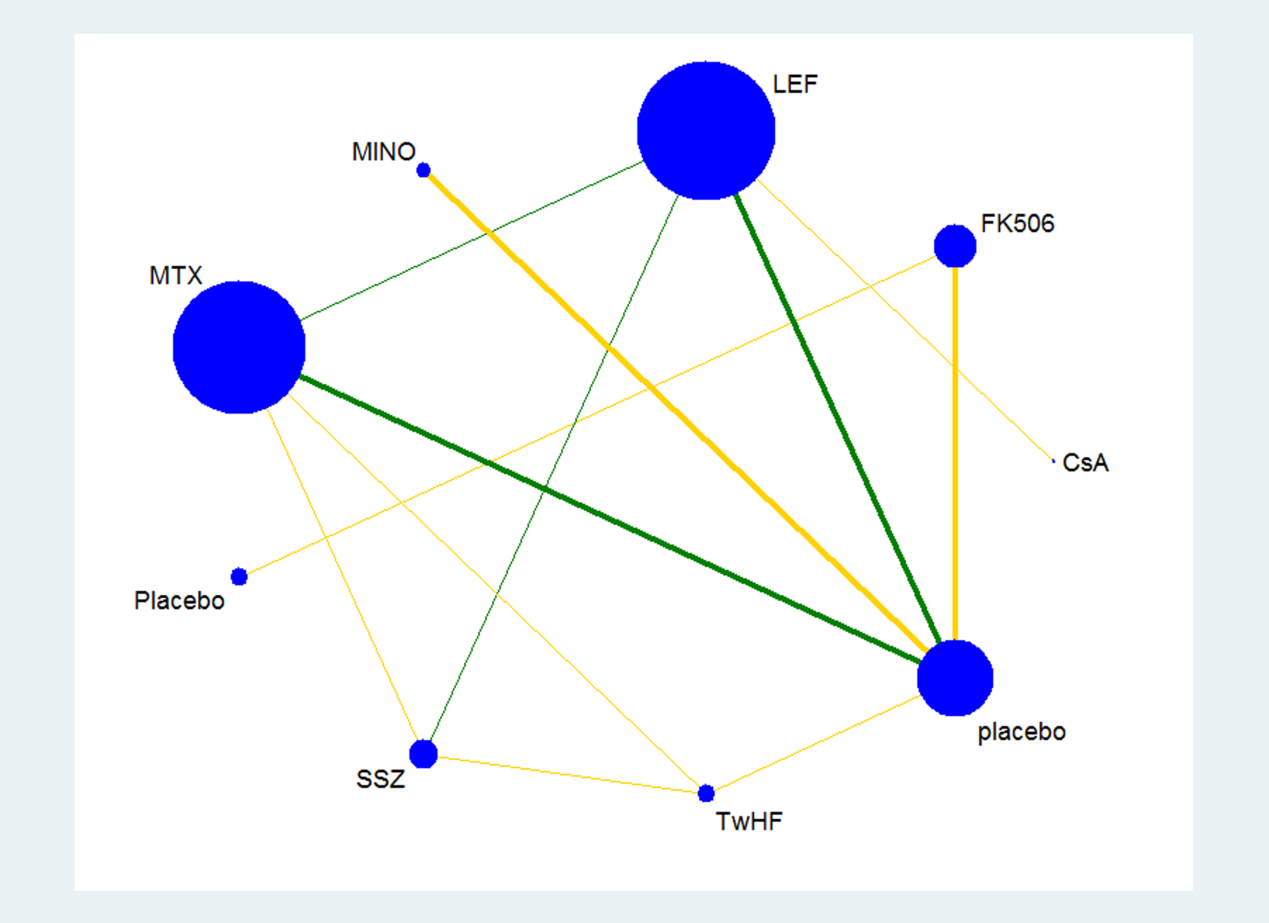
**

**Figure S1C**

**
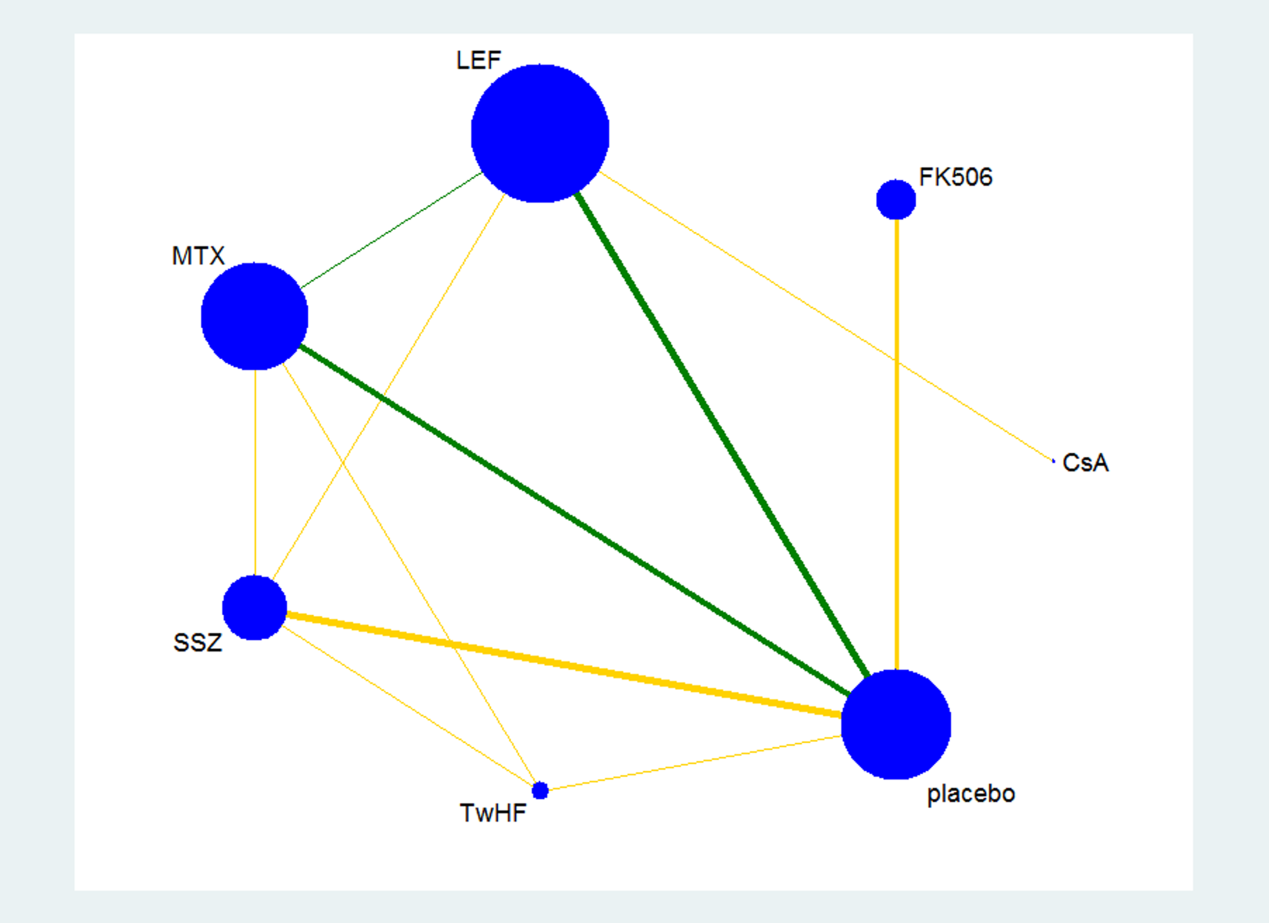
**

**Figure S1D**





**Figure S2**


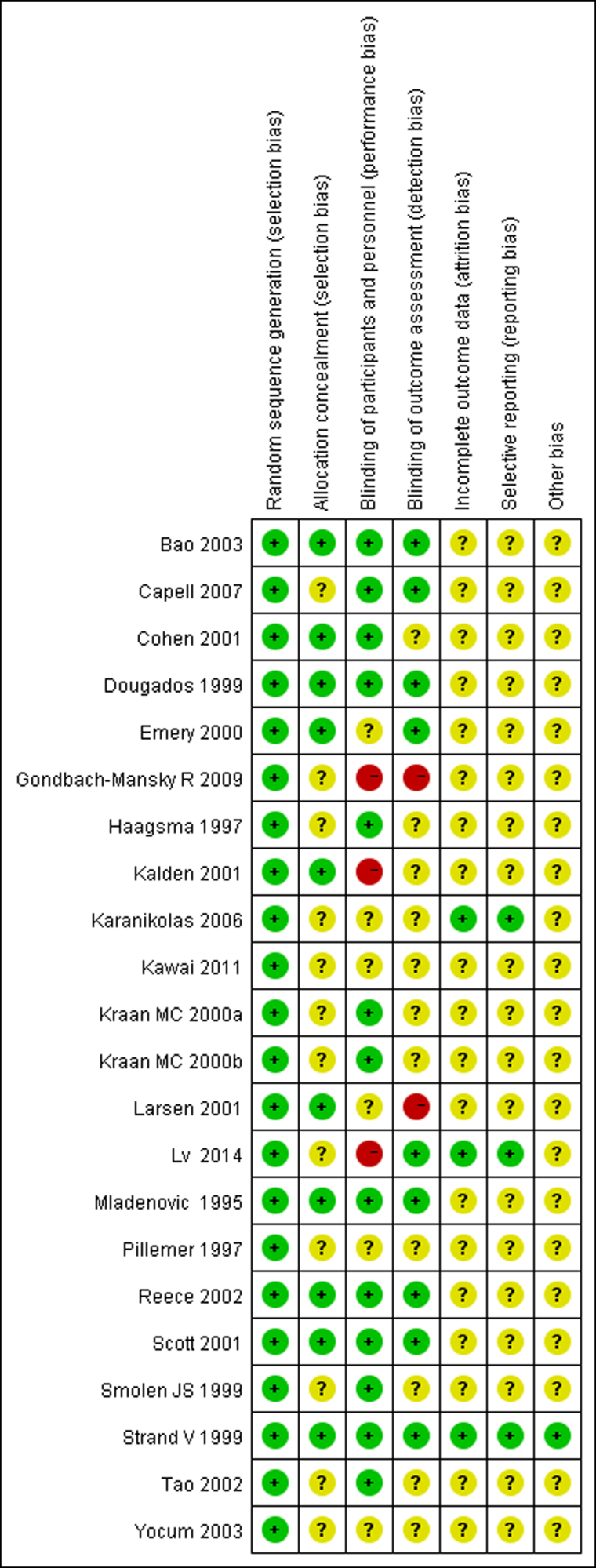


**Figure S3**


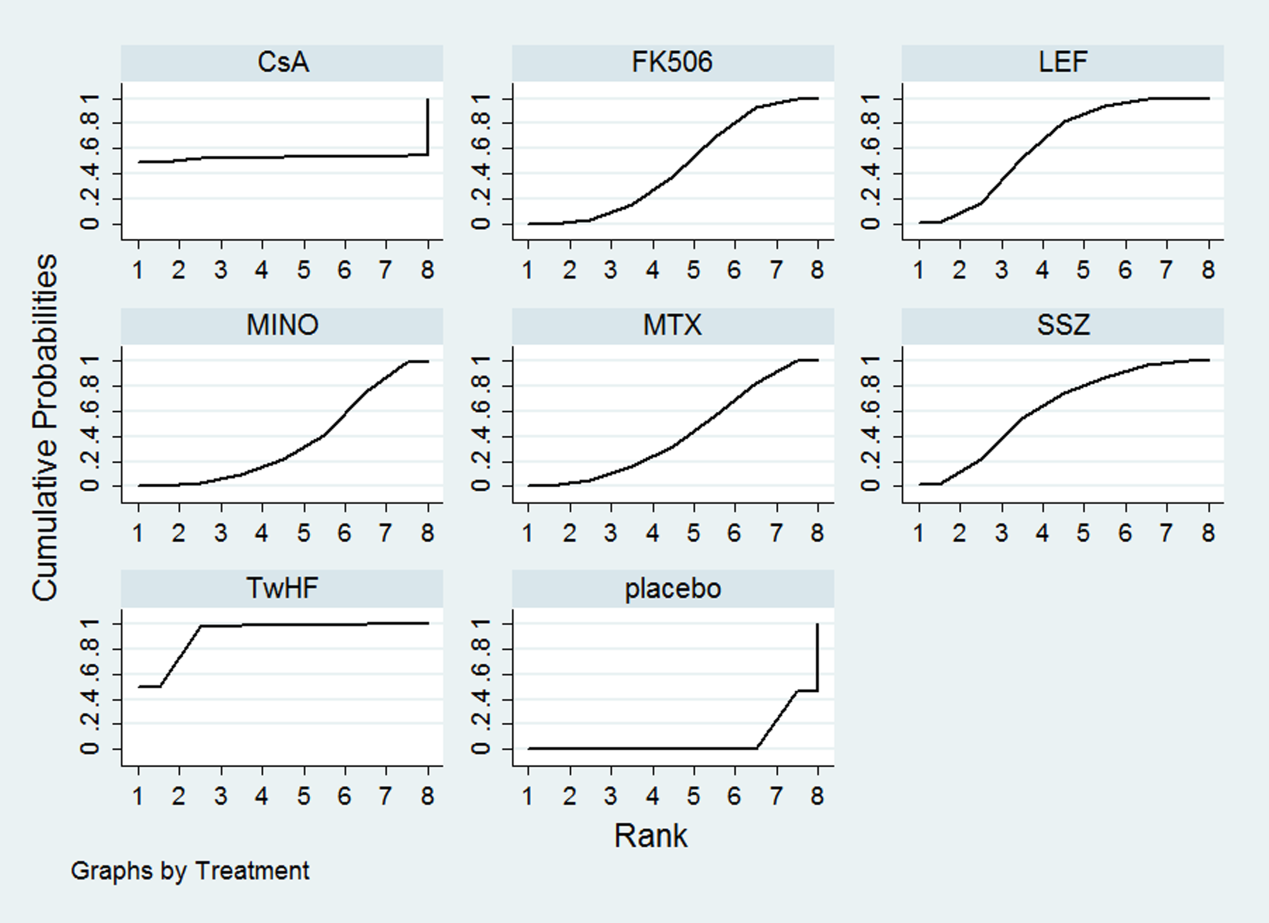


**Figure S4A**


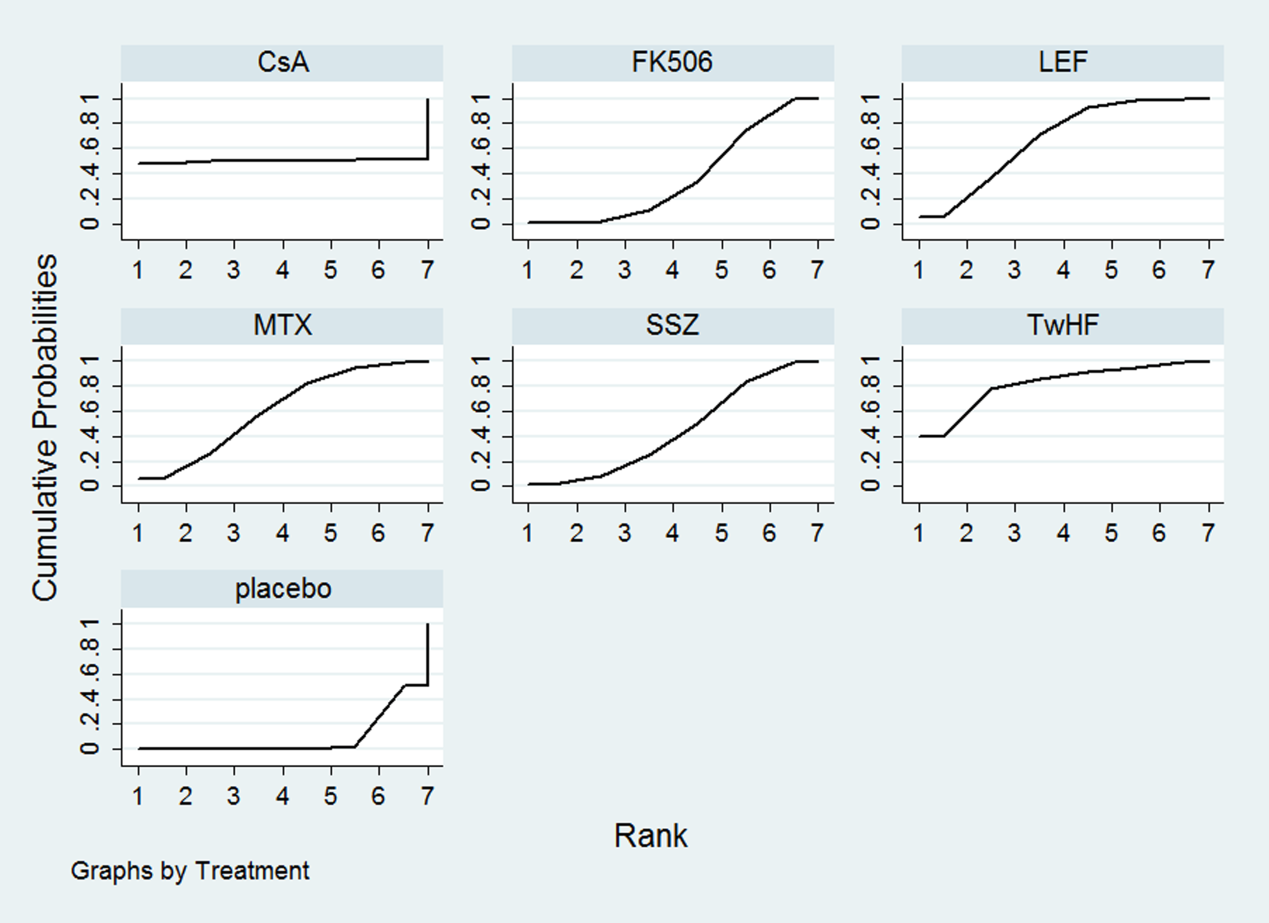


**Figure S4B**

**
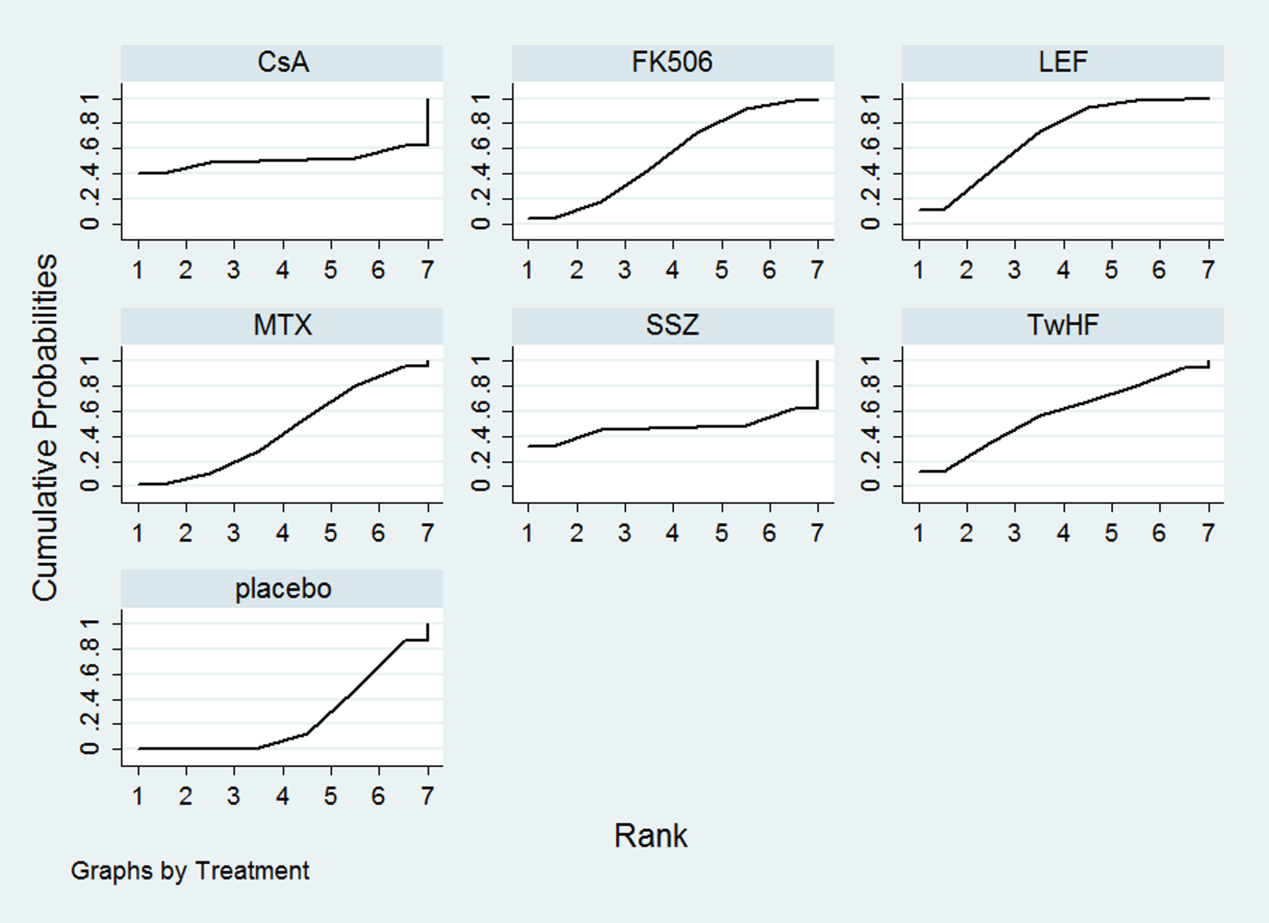
**

**Figure S4C**


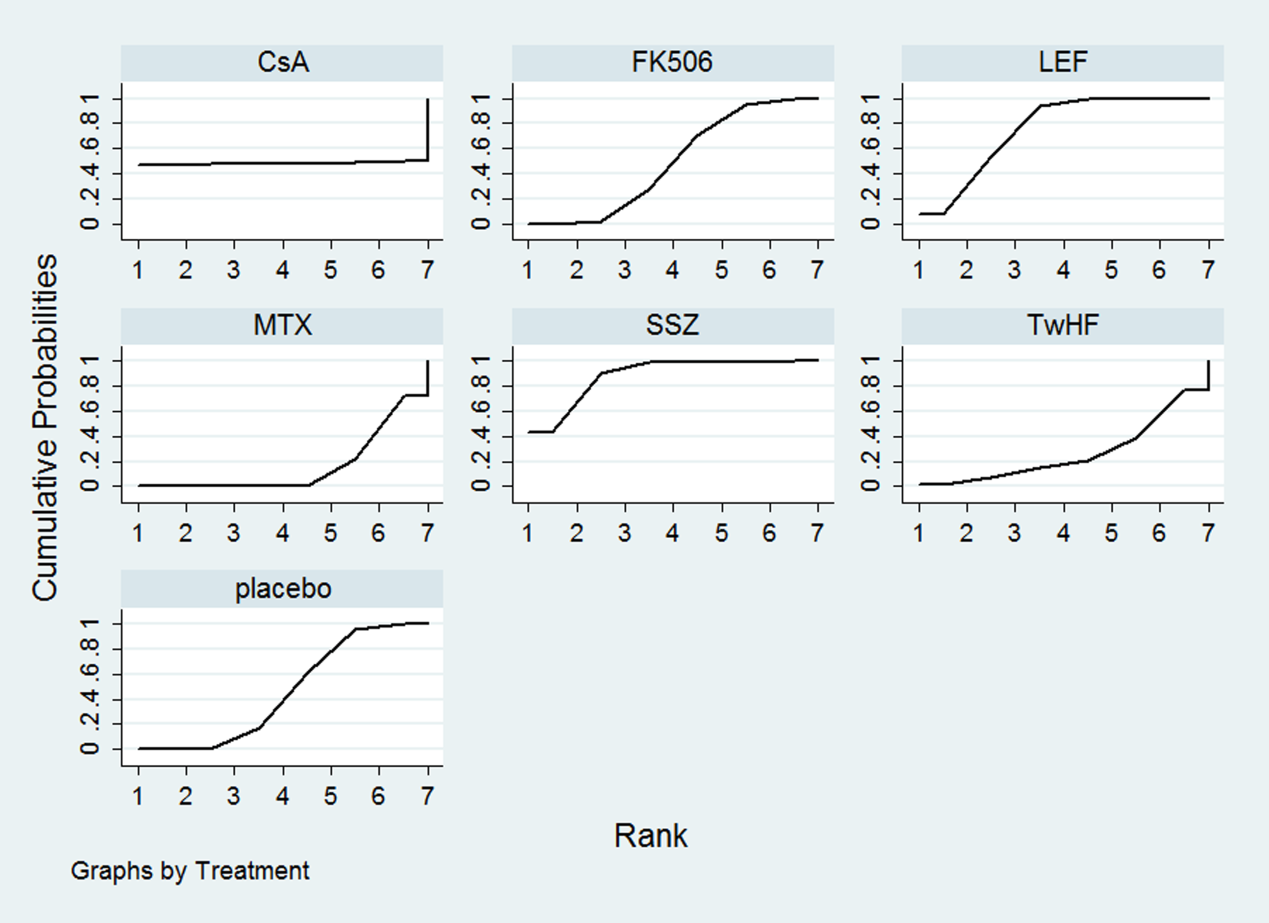


**Figure S4D**

**
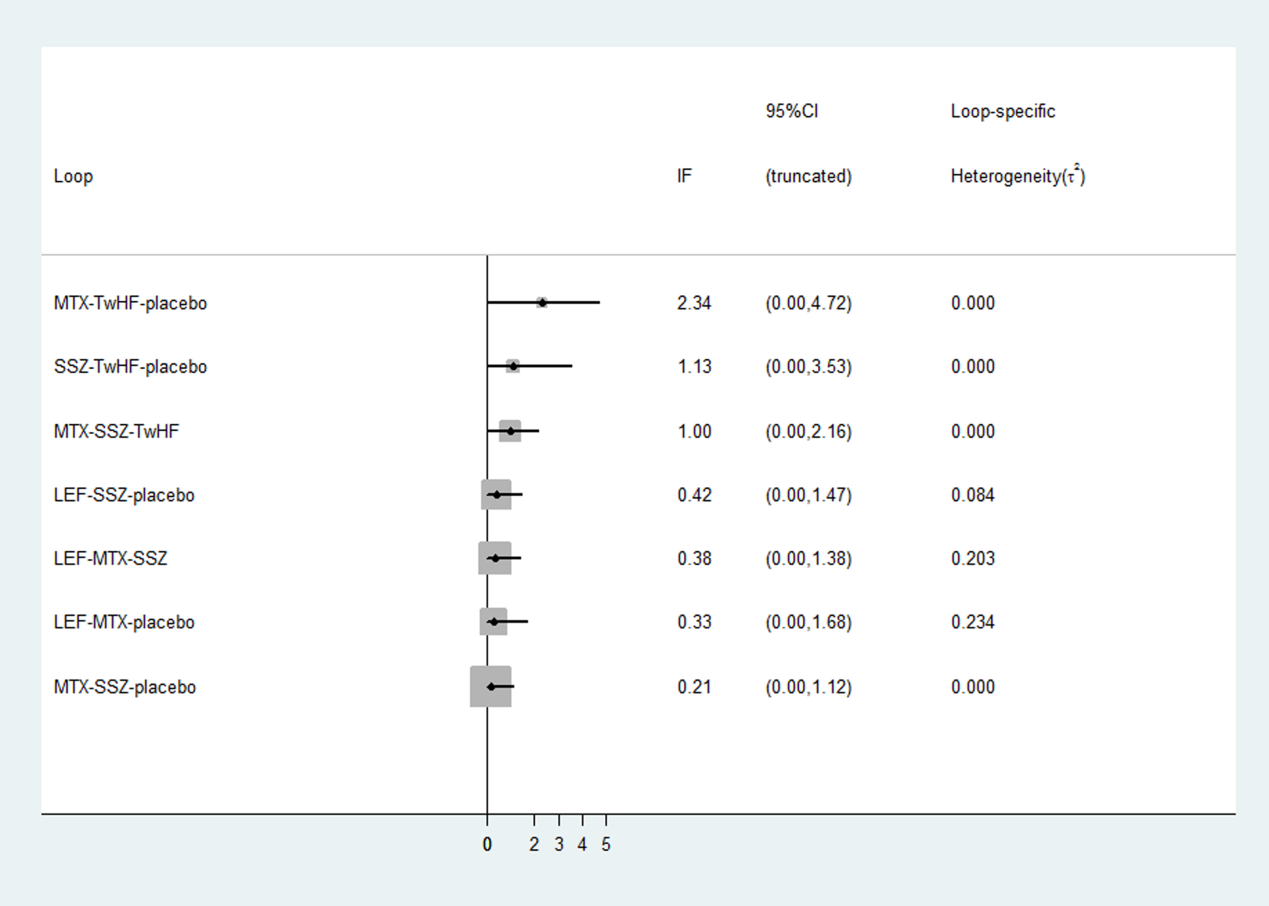
**

**Figure S5A**

**
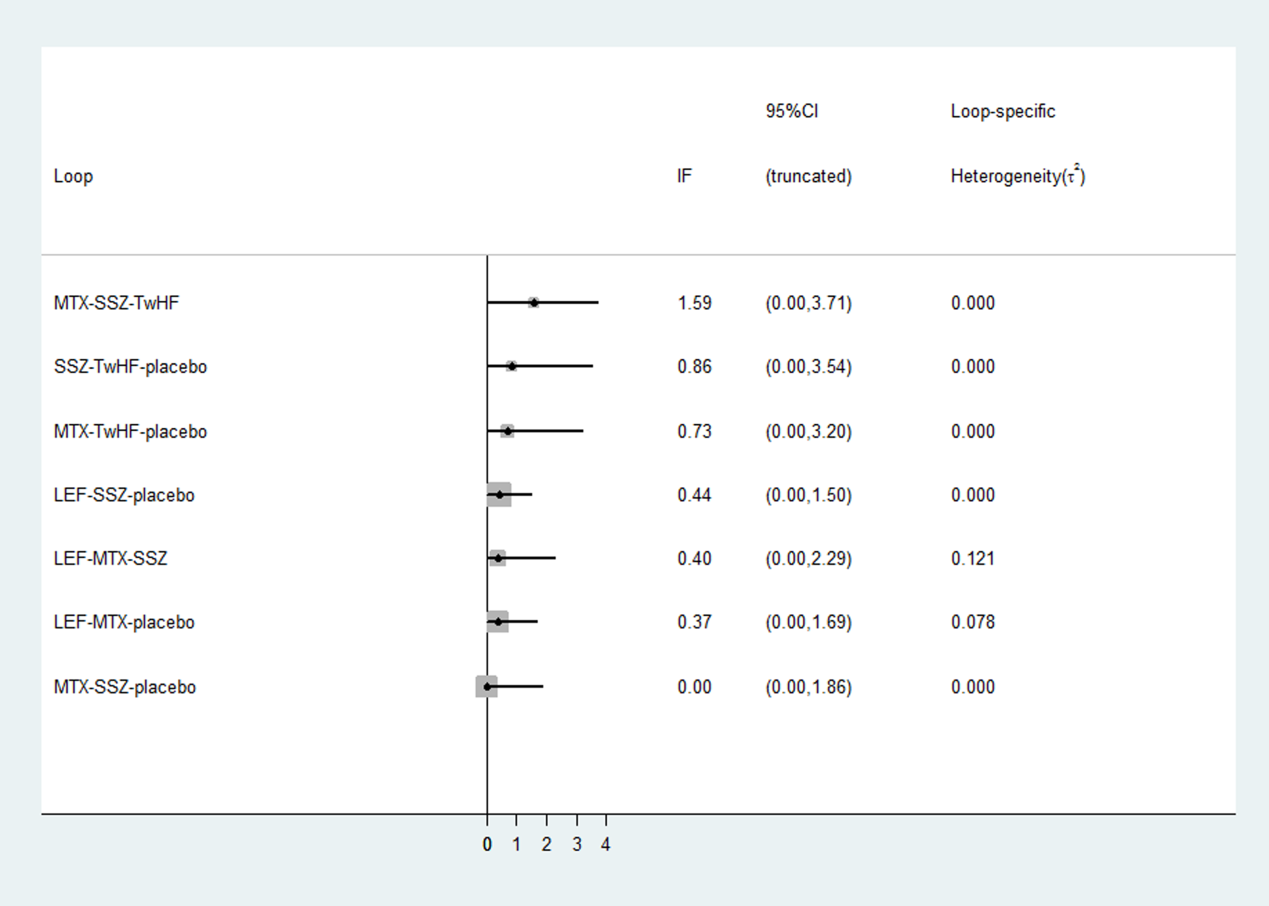
**

**Figure S5B**

**
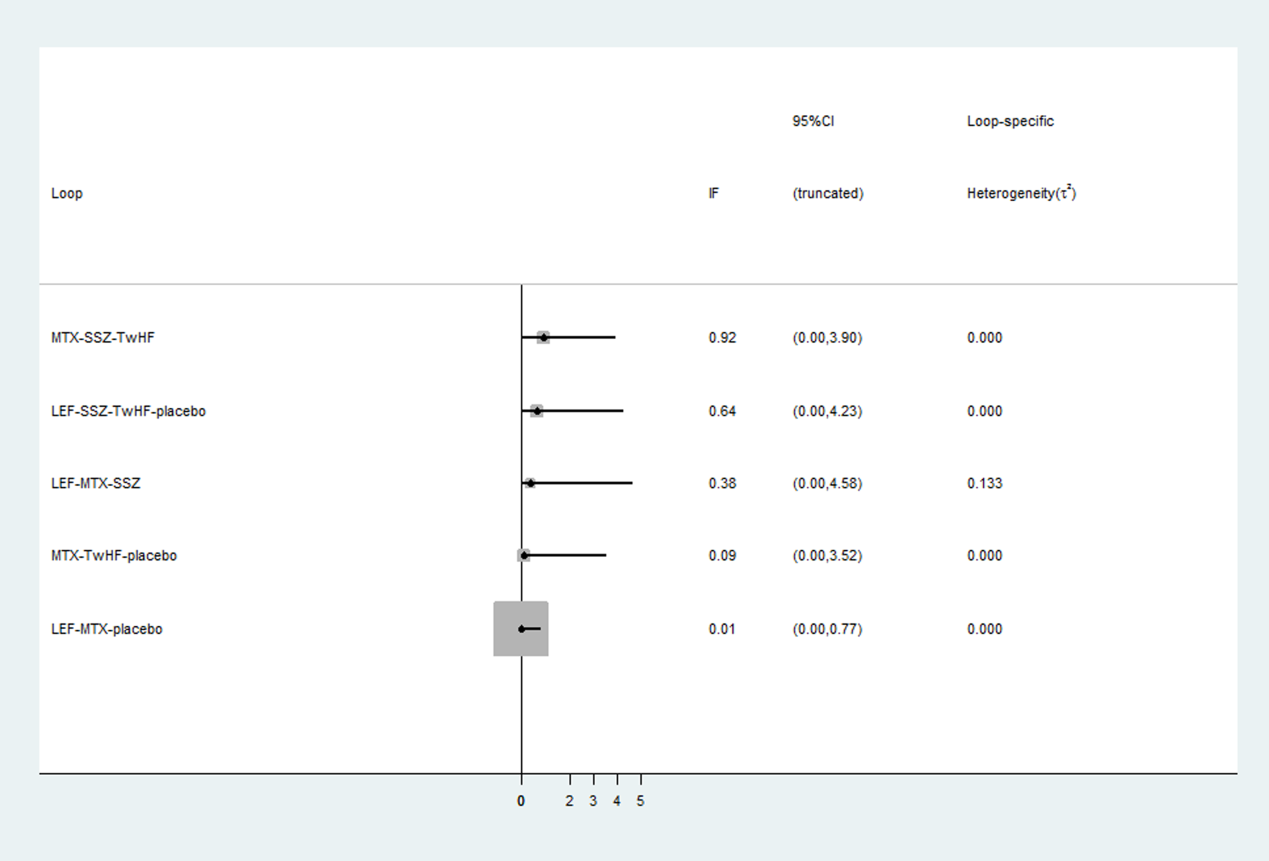
**

**Figure S5C**

**
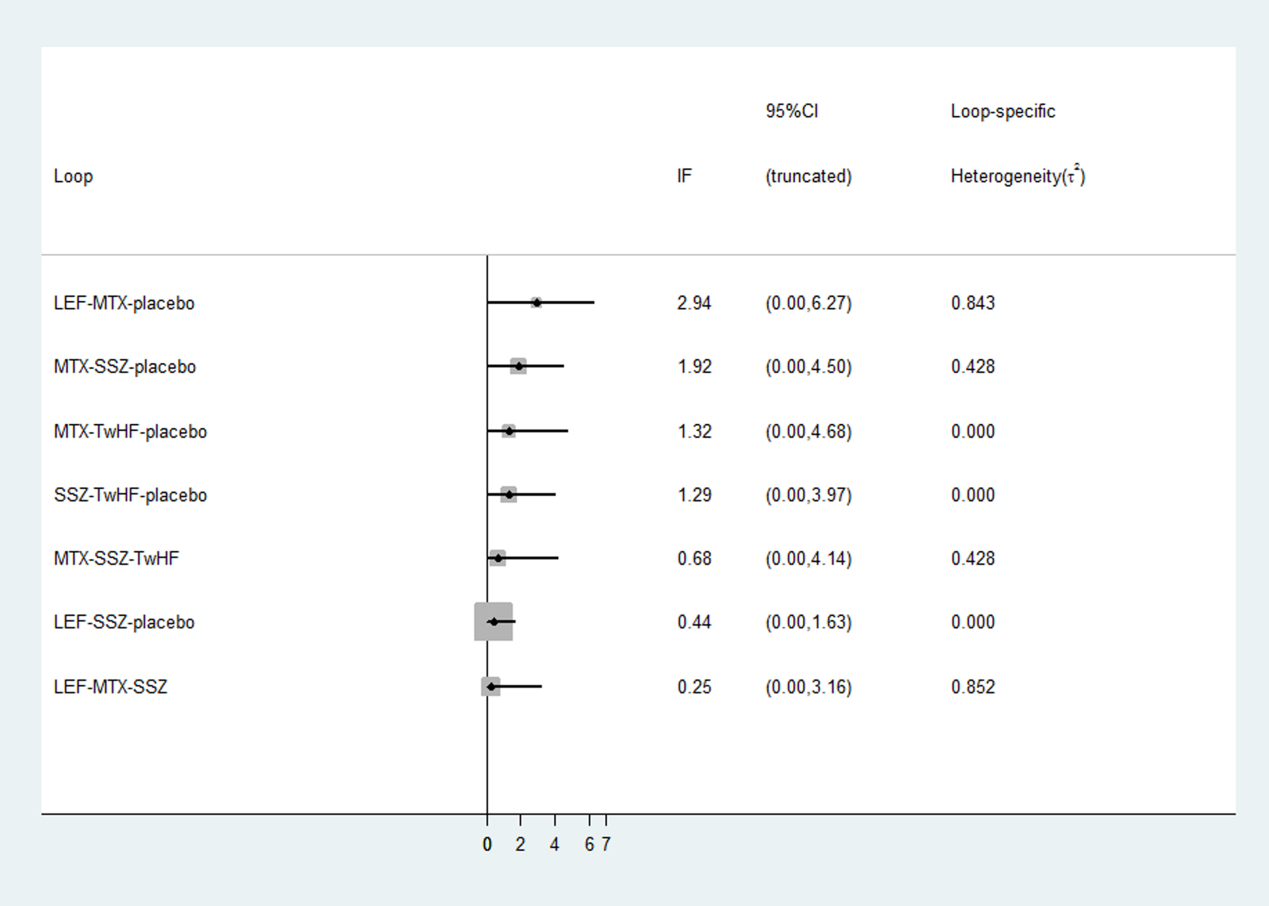
**

**Figure S5D**

**
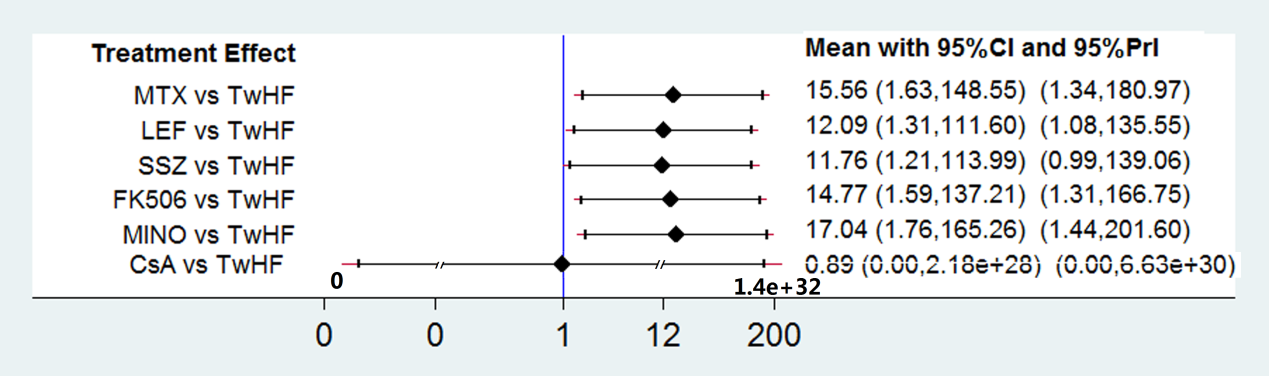
**

**Figure S6A**

**
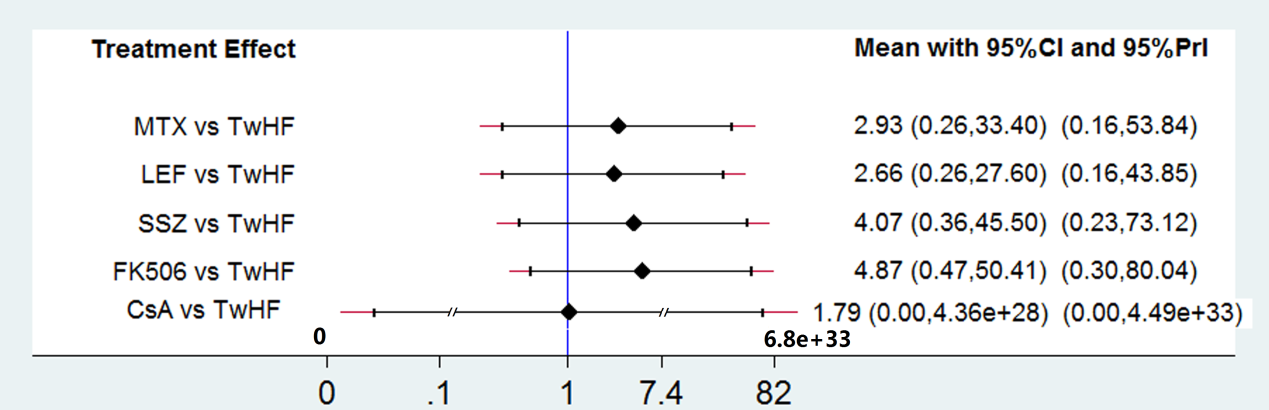
**

**Figure S6B**

**
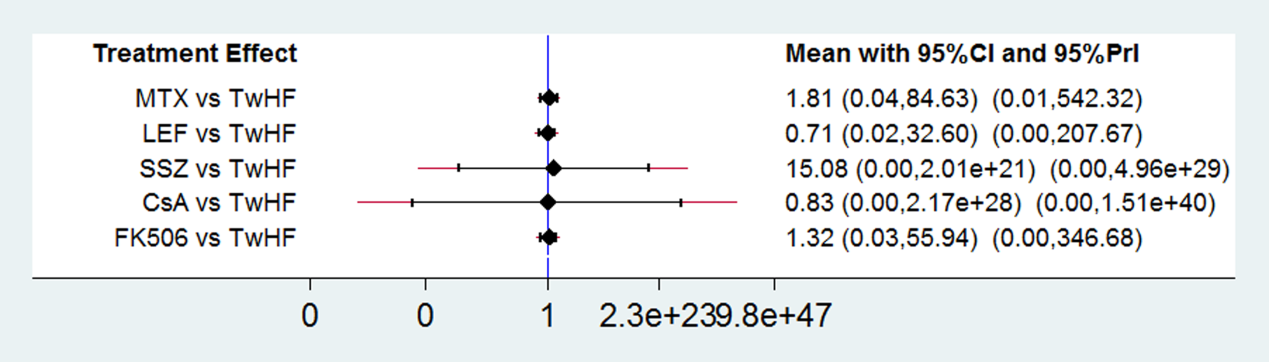
**

**Figure S6C**

**
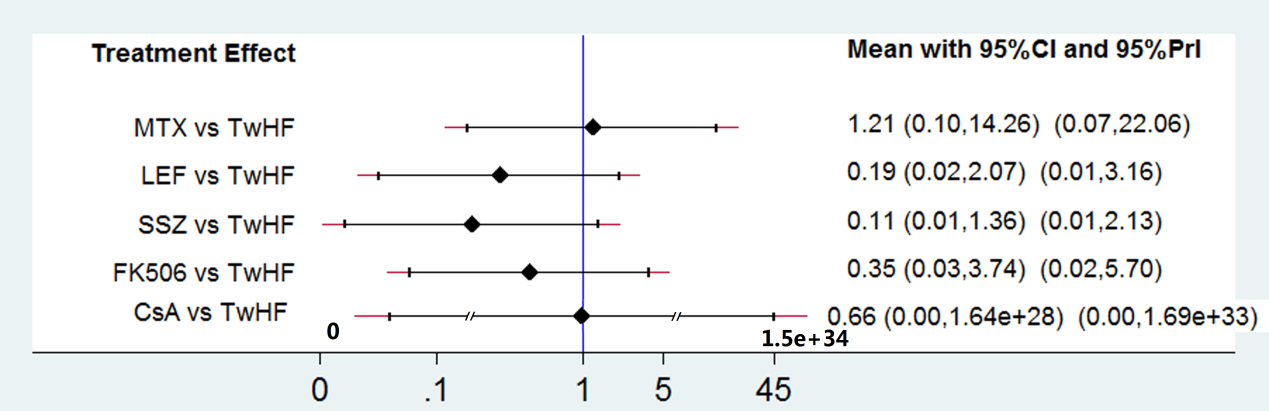
**

**Figure S6D**

**
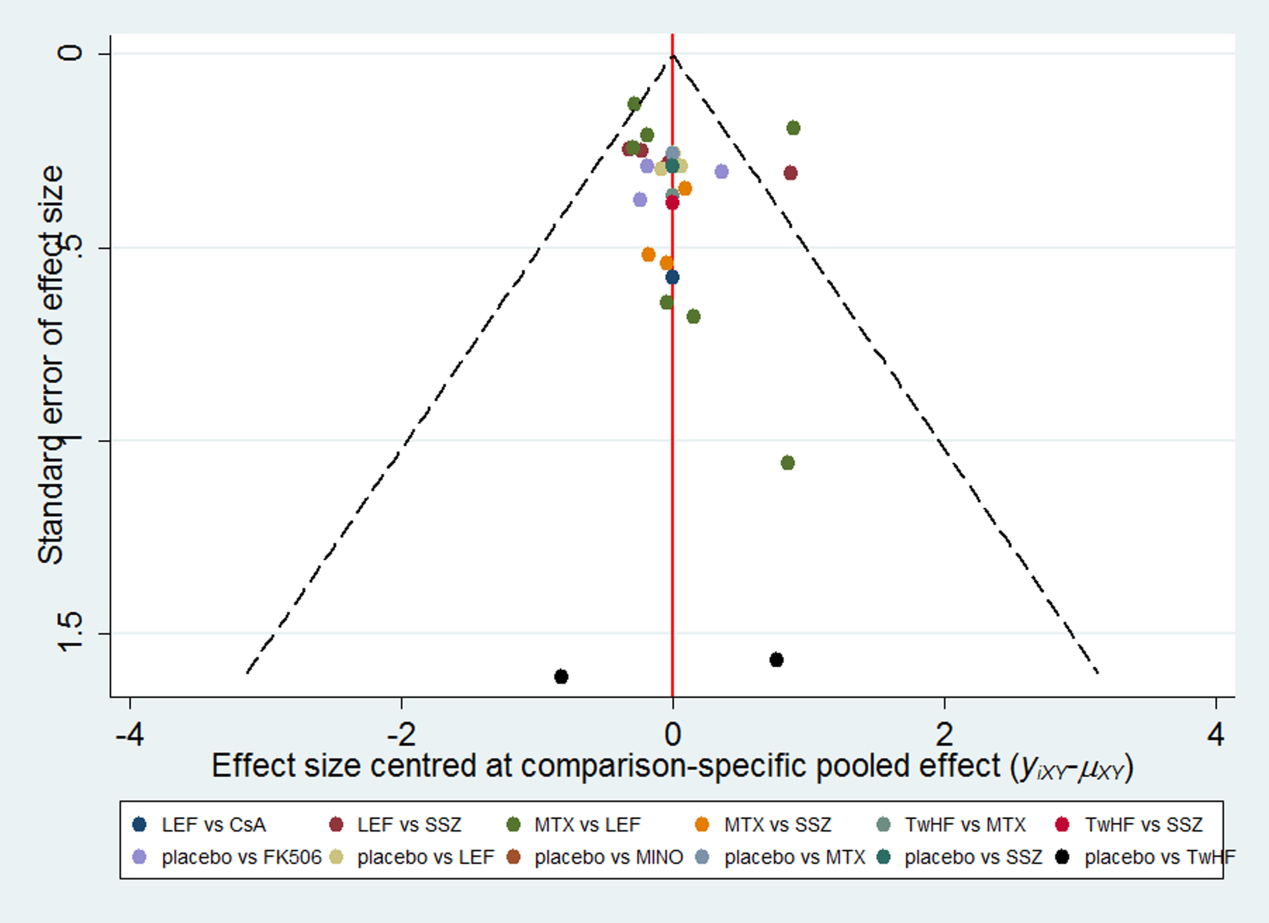
**

**Figure S7A**

**
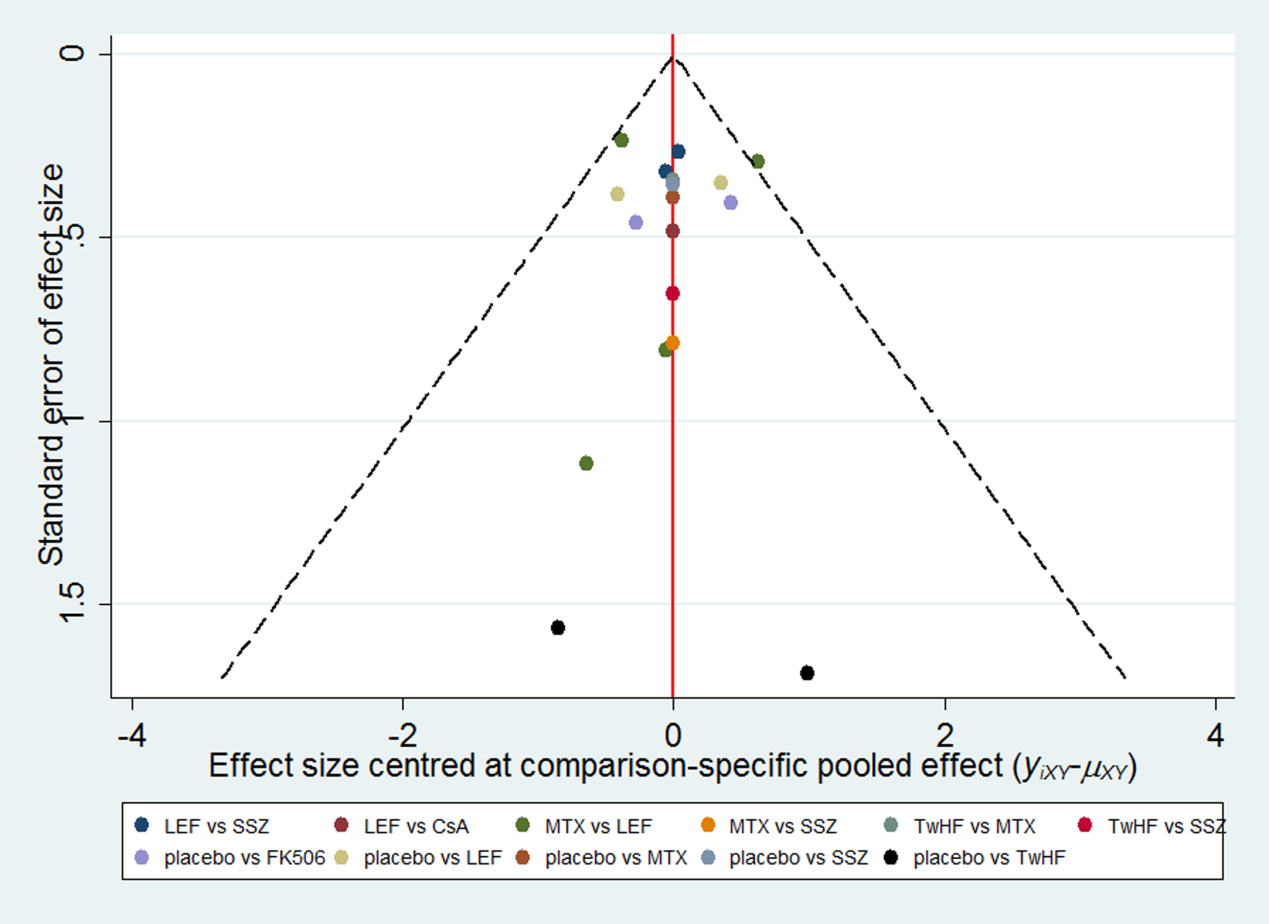
**

**Figure S7B**

**
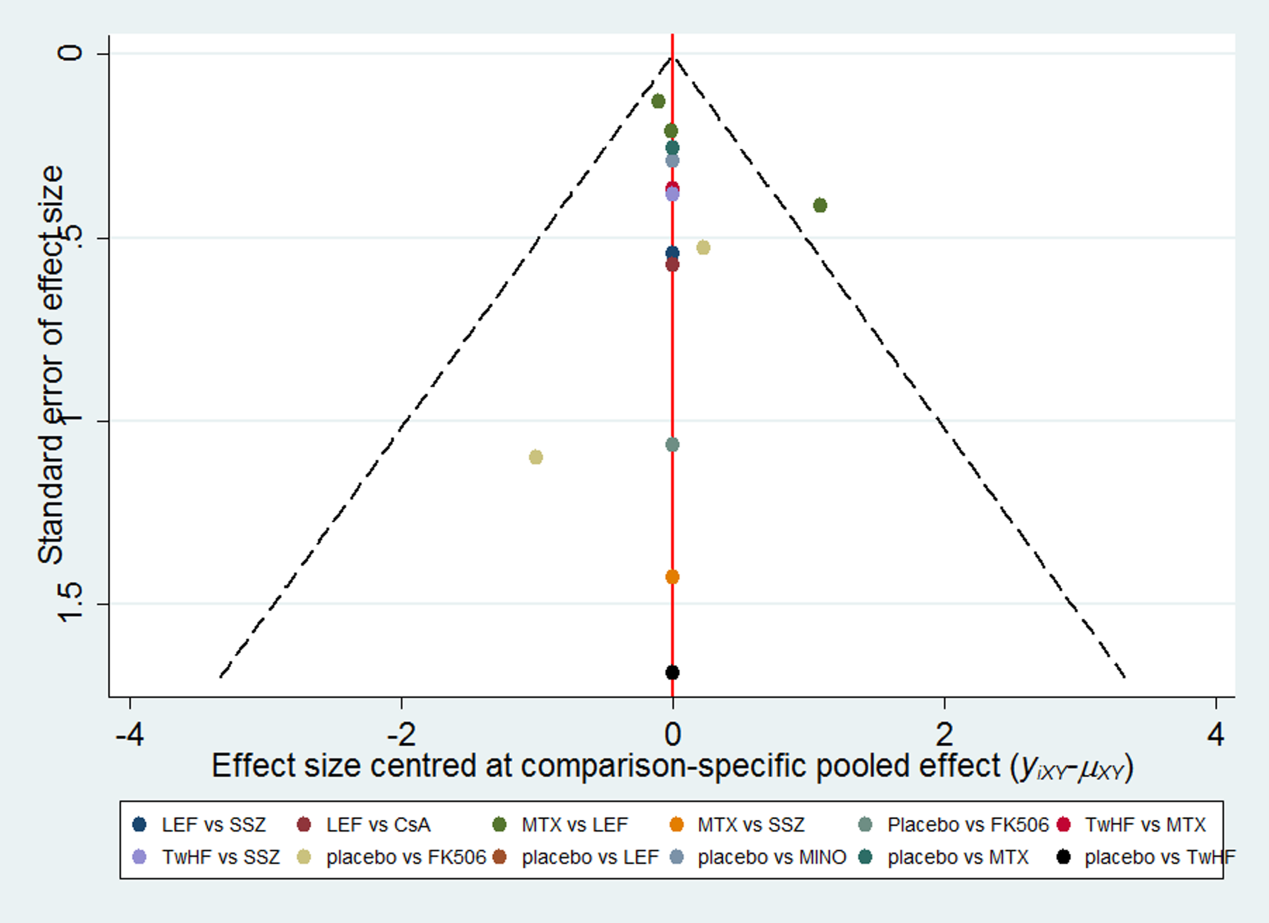
**

**Figure S7C**

**
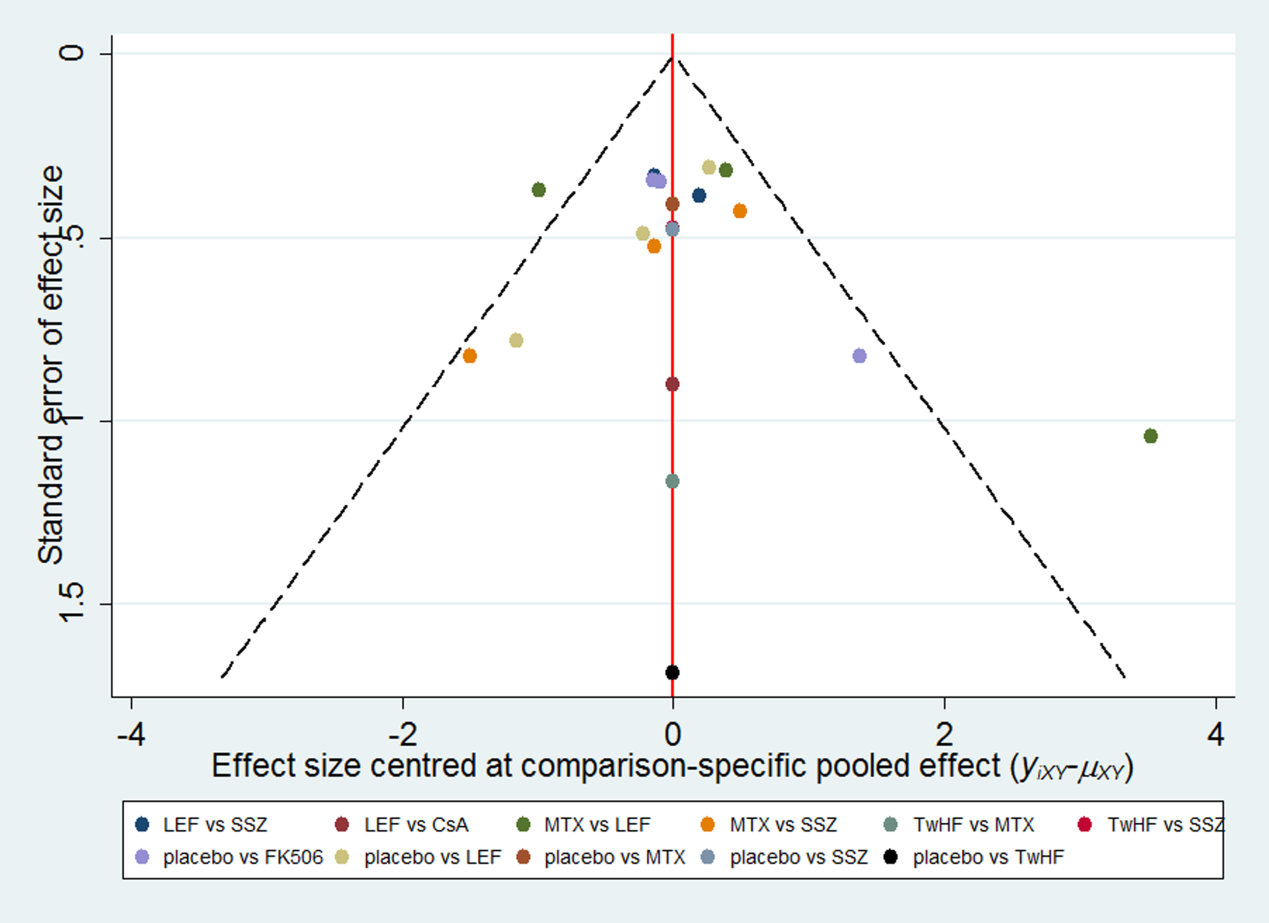
**

**Figure S7D**
