# Supplementary material for: Tripterygium wilfordii Hook F versus conventional synthetic disease-modifying anti-rheumatic drugs as monotherapy for rheumatoid arthritis: a systematic review and network meta-analysis
Source: BMC Complement Altern Med. 2016 Jul 13;16:215. doi: 10.1186/s12906-016-1194-x (PMC4944439; doi:10.1186/s12906-016-1194-x)
Supplement: Additional file 3: Table S1. — Baseline characteristics of included trials (Additional file 4). Table S2. ACR 50 by direct comparisons and network meta-analysis. Table S3. ACR 70 by direct comparisons and network meta-analysis. (DOCX 32 kb) [file 12906_2016_1194_MOESM3_ESM.docx]

**Table S1** Baseline characteristics of included trials

| **Trial** | **No. and location of centers** | **Compared interventions** | **Endpoints** | **Sample size** | **Study period** | **RF status (% positive)** | **Gender (%F)** | **Mean age (years)** | **Duration since diagnosis** |
| --- | --- | --- | --- | --- | --- | --- | --- | --- | --- |
| **Lv, 2015[1]** | 9; China | TwHF | ACR 20, 50, 70, safety | 69 | 24 weeks | 81.2 | 81.2 | 51.3 | 61.9 |
|  |  | MTX |  | 69 |  | 76.8 | 85.5 | 51 | 58.8 |
| **Goldbach-Mansky R, 2009[2]** | 11; United States | TwHF | ACR 20, 50, 70; safety | 60 | 24 weeks | - | 73 | 54 | - |
|  |  | SSZ |  | 61 |  | - | 87 | 52 | - |
| **Tao, 2002[3]** | 1; United States | TwHF_1_ | ACR 20, safety; | 10 | 20 weeks | - | 66.7 | 54 | 14 |
|  |  | TwHF_2_ |  | 10 |  | - | 72.7 | 57 | 20 |
|  |  | Placebo |  | 12 |  | - | 100 | 51 | 20 |
| **Strand V, 1999[4]** | 42; United States, Canada | MTX | ACR 20, 50, 70, safety | 182 | 52 weeks | 59.4 | 75.3 | 53.3 | 78 |
|  |  | LEF |  | 182 |  | 64.8 | 72.5 | 54.1 | 84 |
|  |  | Placebo |  | 118 |  | 60.2 | 70.3 | 54.6 | 83 |
| **Emery P, 2000 [5]** | 117; Europe, South Africa | MTX | ACR 20 | 498 | 12 weeks | - | 71.3 | 57.8 | 46 |
|  |  | LEF |  | 501 |  | - | 70.7 | 58.3 | 44 |
| **Kraan MC, 2000[6]** | 2; The Netherlands, United Kingdom | MTX | ACR 20, 50 | 19 | 16 weeks | - | - | - | - |
|  |  | LEF |  | 16 |  | - | - | - | - |
| **Kraan MC, 2000[7]** | 2; The Netherlands | MTX | ACR 20, 50 | 8 | 16 and 52 weeks | - | 37.5 | 66 | 133 |
|  |  | LEF |  | 7 |  | - | 57.1 | 63 | 33 |
| **Bao, 2003[8]** | 9; China | MTX | ACR 20, safety | 213 | 24 weeks | - | 79.8 | 45.81 | 46.31 |
|  |  | LEF |  | 291 |  | - | 81.1 | 46.59 | 58.82 |
| **Reece, 2002[9]** | 1; United Kingdom | MTX | ACR20 | 21 | 4 months | - | - | - |  |
|  |  | LEF |  | 18 |  | - | - | - |  |
| **Cohen, 2001[10]** | 1; United States | MTX | ACR, 2050, 70, safety | 190 | 12months | - | 74 | 53 | 78 |
|  |  | LEF |  | 190 |  | - | 73 | 54 | 83 |
| **Capell, 2007[11]** | 8; Glasgow, Lanarkshire, Inverness | MTX | ACR20, 50, 70, safety | 54 | 6-month | 65 | 79 | 53 | 12 |
|  |  | SSZ |  | 55 |  | 64 | 75 | 55 | 12 |
| **Haagsma, 1997[12]** | 1; The netherlands | MTX | ACR20, safety | 35 | 52 weeks | 94.3 | 65.7 | 54.9 | 3 |
|  |  | SSZ |  | 34 |  | 97.1 | 61.8 | 56.8 | 3.1 |
| **Dougados 1999[13]** | 3; Finland, France, Germany | MTX | ACR20, safety | 69 | 52 weeks | 62 | 74 | 50 | 18.4 |
|  |  | SSZ |  | 68 |  | 75 | 71 | 52 | 10.8 |
| **Mladenovic V, 1995 [14]** | 6; Yugoslavia, Croatia, Slovenia | LEF | ACR 20, safety; | 101 | 24 weeks | 73.67 | 85.67 | 50.6 | 101 |
|  |  | Placebo |  | 102 |  | 76.47 | 75.49 | 52.8 | 100 |
| **Smolen JS, 1999[15]** | 36; Europe, Australia, New Zealand, South Africa | LEF | ACR 20, 50, safety | 133 | 104 weeks | 79 | 76 | 58.3 | 91 |
|  |  | SSZ |  | 133 |  | 80 | 69 | 58.9 | 89 |
|  |  | Placebo |  | 92 |  | 83 | 75 | 58.8 | 68 |
| **Kalden, 2001[16]** | Australia, Europe, New Zealand, South Africa | LEF | ACR20 | 116 | 12mnths | - | 76 | 58 | 96 |
|  |  | SSZ |  | 113 |  | - | 69 | 59 | 84 |
| **Karanikolas, 2006[17]** | 2; Greece | LEF | ACR20, 50, 70, safety | 36 | 12 months | - | - | - | - |
|  |  | CsA |  | 35 |  | - | - | - | - |
| **Larsen, 2001[18]** | Astralia, Europe, Eew Zealand, and South Africa | LEF | ACR20, safety | 133 | 12months | - | 76 | 58 | 96 |
|  |  | SSZ |  | 133 |  | - | 69 | 59 | 84 |
| **Scott, 2001[19]** | Astralia, Europe, Eew Zealand, and South Africa | LEF | ACR20, 50, 70 | 130 | 6months | - | 76 | 58 | 96 |
|  |  | SSZ |  | 132 |  | - | 69 | 59 | 84 |
| **Yocum, 2003[20]** | 54; United States | FK506_1_ | ACR20, 50, 70, safety | 154 | 6 months | 75.3 | 76.0 | 55.9 | 11.4 |
|  |  | FK506_2_ |  | 153 |  | 65.4 | 78.4 | 55.8 | 11.3 |
|  |  | Placebo |  | 157 |  | 63.1 | 75.8 | 55.8 | 11.8 |
| **Kawai, 2011[21]** | 32; Japan | FK506 | ACR20, 50, 70, safety | 61 | 28 weeks | - | 55 | 47.1 | 19 |
|  |  | Placebo |  | 62 |  | - | 50 | 50.0 | 20 |
| **Pillemer, 1997[22]** | - | MINO | ACR20 | 109 | 48weeks | - | 76 | 55 | 101 |
|  |  | Placebo |  | 110 |  | - | 80 | 53.5 | 106 |

TwHF: *Tripterygium wilfordii* Hook F, MTX: methotrexate, LEF: leflunomide, SSZ: sulphasalazine, CsA: cyclosporine, FK506: tacrolimus, and MINO: minocycline.

**Table S2** Odds ratio (95%CI) by direct comparison (left lower part) and network meta- analysis (right upper part) based on ACR50

| TwHF | 2.93(0.26-33.38) | 2.66(0.26-27.60) | 4.07(0.36-45.50) | 1.79(0.00-4.36e+28) | 4.87(0.47-50.40) | 11.11(1.09-100.0) |
| --- | --- | --- | --- | --- | --- | --- |
| 1.42(0.73-2.77) | MTX | 0.91(0.32-2.61) | 1.39(0.42-4.66) | 0.61(0.00-1.44e+28) | 1.66(0.58-4.74) | 3.57(1.49-9.09) |
| - | 0.83(0.60-1.15) | LEF | 1.53(0.56-4.19) | 0.67(0.00-1.58e+28) | 1.83(0.82-4.11) | 4.00(2.08-7.14) |
| 9.67(2.69-34.72) | 1.39(0.30-6.51) | 1.07(0.72-1.59) | SSZ | 0.44(0.00-1.04e+28) | 1.20(0.44-3.25) | 2.63(1.15-5.88) |
| - | - | 1.07(0.42-2.76) | - | CsA | 2.72(0.00-6.39e+28) | 5.88(0.00-100.0) |
| - | - | - | - | - | FK506 | 2.17(1.25-3.85) |
| 21.97(1.15-419.2) | 3.63(1.70-7.79) | 4.27(2.59-7.04) | 2.61(1.31-5.23) | - | 3.11(1.98-4.91) | Placebo |

Results of direct comparisons are listed in the lower-left triangle, and the estimation is calculated as the row-defining treatment compared with the column-defining treatment. Results of network meta-analysis are listed in the upper-right triangle, and the estimation is calculated as the column-defining treatment compared with the row-defining treatment..

TwHF: *Tripterygium wilfordii* Hook F, MTX: methotrexate, LEF: leflunomide, SSZ: sulphasalazine, CsA: cyclosporine, FK506: tacrolimus.

**Table S3** Odds ratio (95%CI) by direct comparison (left lower part) and network meta- analysis (right upper part) based on ACR70

| TwHF | 1.81(0.04-84.63) | 0.71(0.02-32.60) | 15.08(0.00-2.01e+21) | 0.83(0.00-2.17e+28) | 1.32(0.03-55.94) | 4.00(0.12-100.00) |
| --- | --- | --- | --- | --- | --- | --- |
| 1.50(0.73-3.08) | MTX | 0.39(0.04-3.53) | 8.34(0.00-1.00e+21) | 0.46(0.00-1.11e+28) | 0.73(0.09-5.70) | 2.17(0.45-10.00) |
| - | 0.84(0.70-1.01) | LEF | 21.27(0.00-2.55e+21) | 1.17(0.00-2.83e+28) | 1.85(0.24-14.13) | 5.56(1.20-25.00) |
| 12(1.49-96.98) | 0.98(0.06-16.10) | 1.56(0.54-4.52) | SSZ | 0.06(0.00-3.13e+33) | 0.09(0.00-1.04e+19) | 0.26(0.00-100.0) |
| - | - | 0.47(0.15-1.46) | - | CsA | 1.58(0.00-3.79e+40) | 4.76(0.00-100.0) |
| - | - | - | - | - | FK506 | 3.03(0.79-11.11) |
| 3.95(0.14-108.1) | 2.18(0.78-6.12) | 3.07(1.85-5.07) | - | - | 2.71(1.24-5.94) | Placebo |

Results of direct comparisons are listed in the lower-left triangle, and the estimation is calculated as the row-defining treatment compared with the column-defining treatment. Results of network meta-analysis are listed in the upper-right triangle, and the estimation is calculated as the column-defining treatment compared with the row-defining treatment..

TwHF: *Tripterygium wilfordii* Hook F, MTX: methotrexate, LEF: leflunomide, SSZ: sulphasalazine, CsA: cyclosporine, FK506: tacrolimus.
