# Supplementary material for: Tripterygium wilfordii Hook F versus conventional synthetic disease-modifying anti-rheumatic drugs as monotherapy for rheumatoid arthritis: a systematic review and network meta-analysis
Source: BMC Complement Altern Med. 2016 Jul 13;16:215. doi: 10.1186/s12906-016-1194-x (PMC4944439; doi:10.1186/s12906-016-1194-x)
Supplement: Additional file 4: — Appendix Information Methods of network meta-analysis in the study. (DOC 34 kb) [file 12906_2016_1194_MOESM4_ESM.doc]

**Appendix Reference of Table S1**

1. Lv QW, Zhang W, Shi Q, Zheng WJ, Li X, Chen H, Wu QJ, Jiang WL, Li HB, Gong L *et al*: **Comparison of Tripterygium wilfordii Hook F with methotrexate in the treatment of active rheumatoid arthritis (TRIFRA): a randomised, controlled clinical trial**. *Annals of the rheumatic diseases* 2015, **74**(6):1078-1086.

2. Goldbach-Mansky R, Wilson M, Fleischmann R, Olsen N, Silverfield J, Kempf P, Kivitz A, Sherrer Y, Pucino F, Csako G *et al*: **Comparison of Tripterygium wilfordii Hook F versus sulfasalazine in the treatment of rheumatoid arthritis: a randomized trial**. *Annals of internal medicine* 2009, **151**(4):229-240, W249-251.

3. Tao X, Younger J, Fan FZ, Wang B, Lipsky PE: **Benefit of an extract of Tripterygium Wilfordii Hook F in patients with rheumatoid arthritis: a double-blind, placebo-controlled study**. *Arthritis and rheumatism* 2002, **46**(7):1735-1743.

4. Strand V, Cohen S, Schiff M, Weaver A, Fleischmann R, Cannon G, Fox R, Moreland L, Olsen N, Furst D *et al*: **Treatment of active rheumatoid arthritis with leflunomide compared with placebo and methotrexate. Leflunomide Rheumatoid Arthritis Investigators Group**. *Archives of internal medicine* 1999, **159**(21):2542-2550.

5. Emery P, Breedveld FC, Lemmel EM, Kaltwasser JP, Dawes PT, Gomor B, Van Den Bosch F, Nordstrom D, Bjorneboe O, Dahl R *et al*: **A comparison of the efficacy and safety of leflunomide and methotrexate for the treatment of rheumatoid arthritis**. *Rheumatology* 2000, **39**(6):655-665.

6. Kraan MC, Reece RJ, Barg EC, Smeets TJ, Farnell J, Rosenburg R, Veale DJ, Breedveld FC, Emery P, Tak PP: **Modulation of inflammation and metalloproteinase expression in synovial tissue by leflunomide and methotrexate in patients with active rheumatoid arthritis. Findings in a prospective, randomized, double-blind, parallel-design clinical trial in thirty-nine patients at two centers**. *Arthritis and rheumatism* 2000, **43**(8):1820-1830.

7. Kraan MC, de Koster BM, Elferink JG, Post WJ, Breedveld FC, Tak PP: **Inhibition of neutrophil migration soon after initiation of treatment with leflunomide or methotrexate in patients with rheumatoid arthritis: findings in a prospective, randomized, double-blind clinical trial in fifteen patients**. *Arthritis and rheumatism* 2000, **43**(7):1488-1495.

8. Bao C, Chen S, Gu Y, Lao Z, Ni L, Yu Q, Xu J, Li X, Liu J, Sun L *et al*: **Leflunomide, a new disease-modifying drug for treating active rheumatoid arthritis in methotrexate-controlled phase II clinical trial**. *Chinese medical journal* 2003, **116**(8):1228-1234.

9. Reece RJ, Kraan MC, Radjenovic A, Veale DJ, O'Connor PJ, Ridgway JP, Gibbon WW, Breedveld FC, Tak PP, Emery P: **Comparative assessment of leflunomide and methotrexate for the treatment of rheumatoid arthritis, by dynamic enhanced magnetic resonance imaging**. *Arthritis and rheumatism* 2002, **46**(2):366-372.

10. Cohen S, Cannon GW, Schiff M, Weaver A, Fox R, Olsen N, Furst D, Sharp J, Moreland L, Caldwell J *et al*: **Two-year, blinded, randomized, controlled trial of treatment of active rheumatoid arthritis with leflunomide compared with methotrexate. Utilization of Leflunomide in the Treatment of Rheumatoid Arthritis Trial Investigator Group**. *Arthritis and rheumatism* 2001, **44**(9):1984-1992.

11. Capell HA, Madhok R, Porter DR, Munro RA, McInnes IB, Hunter JA, Steven M, Zoma A, Morrison E, Sambrook M *et al*: **Combination therapy with sulfasalazine and methotrexate is more effective than either drug alone in patients with rheumatoid arthritis with a suboptimal response to sulfasalazine: results from the double-blind placebo-controlled MASCOT study**. *Annals of the rheumatic diseases* 2007, **66**(2):235-241.

12. Haagsma CJ, van Riel PL, de Jong AJ, van de Putte LB: **Combination of sulphasalazine and methotrexate versus the single components in early rheumatoid arthritis: a randomized, controlled, double-blind, 52 week clinical trial**. *British journal of rheumatology* 1997, **36**(10):1082-1088.

13. Dougados M, Combe B, Cantagrel A, Goupille P, Olive P, Schattenkirchner M, Meusser S, Paimela L, Rau R, Zeidler H *et al*: **Combination therapy in early rheumatoid arthritis: a randomised, controlled, double blind 52 week clinical trial of sulphasalazine and methotrexate compared with the single components**. *Annals of the rheumatic diseases* 1999, **58**(4):220-225.

14. Mladenovic V, Domljan Z, Rozman B, Jajic I, Mihajlovic D, Dordevic J, Popovic M, Dimitrijevic M, Zivkovic M, Campion G *et al*: **Safety and effectiveness of leflunomide in the treatment of patients with active rheumatoid arthritis. Results of a randomized, placebo-controlled, phase II study**. *Arthritis and rheumatism* 1995, **38**(11):1595-1603.

15. Smolen JS, Kalden JR, Scott DL, Rozman B, Kvien TK, Larsen A, Loew-Friedrich I, Oed C, Rosenburg R: **Efficacy and safety of leflunomide compared with placebo and sulphasalazine in active rheumatoid arthritis: a double-blind, randomised, multicentre trial. European Leflunomide Study Group**. *Lancet* 1999, **353**(9149):259-266.

16. Kalden JR, Scott DL, Smolen JS, Schattenkirchner M, Rozman B, Williams BD, Kvien TK, Jones P, Williams RB, Oed C *et al*: **Improved functional ability in patients with rheumatoid arthritis--longterm treatment with leflunomide versus sulfasalazine. European Leflunomide Study Group**. *The Journal of rheumatology* 2001, **28**(9):1983-1991.

17. Karanikolas G, Charalambopoulos D, Andrianakos A, Antoniades C, Katsilambros N: **Combination of cyclosporine and leflunomide versus single therapy in severe rheumatoid arthritis**. *The Journal of rheumatology* 2006, **33**(3):486-489.

18. Larsen A, Kvien TK, Schattenkirchner M, Rau R, Scott DL, Smolen JS, Rozman B, Westhovens R, Tikly M, Oed C *et al*: **Slowing of disease progression in rheumatoid arthritis patients during long-term treatment with leflunomide or sulfasalazine**. *Scandinavian journal of rheumatology* 2001, **30**(3):135-142.

19. Scott DL, Smolen JS, Kalden JR, van de Putte LB, Larsen A, Kvien TK, Schattenkirchner M, Nash P, Oed C, Loew-Friedrich I *et al*: **Treatment of active rheumatoid arthritis with leflunomide: two year follow up of a double blind, placebo controlled trial versus sulfasalazine**. *Annals of the rheumatic diseases* 2001, **60**(10):913-923.

20. Yocum DE, Furst DE, Kaine JL, Baldassare AR, Stevenson JT, Borton MA, Mengle-Gaw LJ, Schwartz BD, Wisemandle W, Mekki QA *et al*: **Efficacy and safety of tacrolimus in patients with rheumatoid arthritis: a double-blind trial**. *Arthritis and rheumatism* 2003, **48**(12):3328-3337.

21. Kawai S, Takeuchi T, Yamamoto K, Tanaka Y, Miyasaka N: **Efficacy and safety of additional use of tacrolimus in patients with early rheumatoid arthritis with inadequate response to DMARDs--a multicenter, double-blind, parallel-group trial**. *Modern rheumatology / the Japan Rheumatism Association* 2011, **21**(5):458-468.

22. Pillemer SR, Fowler SE, Tilley BC, Alarcon GS, Heyse SP, Trentham DE, Neuner R, Clegg DO, Leisen JC, Cooper SM *et al*: **Meaningful improvement criteria sets in a rheumatoid arthritis clinical trial. MIRA Trial Group. Minocycline in Rheumatoid Arthritis**. *Arthritis and rheumatism* 1997, **40**(3):419-425.
